# Epigenetically facilitated mutational assimilation: epigenetics as a hub within the inclusive evolutionary synthesis

**DOI:** 10.1111/brv.12453

**Published:** 2018-09-24

**Authors:** Etienne Danchin, Arnaud Pocheville, Olivier Rey, Benoit Pujol, Simon Blanchet

**Affiliations:** ^1^ Laboratoire Évolution & Diversité Biologique (EDB UMR 5174) Université de Toulouse Midi‐Pyrénées, CNRS, IRD, UPS. 118 route de Narbonne, Bat 4R1 31062 Toulouse Cedex 9 France; ^2^ Department of Philosophy and Charles Perkins Centre University of Sydney Sydney NSW 2006 Australia; ^3^ CNRS, Station d'Ecologie Théorique et Expérimentale (SETE), UMR5321 09200 Moulis France; ^4^ Université de Perpignan Via Domitia, IHPE UMR 5244, CNRS, IFREMER, Université de Montpellier F‐66860 Perpignan France

**Keywords:** non‐genetic inheritance, inclusive heritability, epigenetics, central dogma, genetic assimilation, mutation randomness, inclusive evolutionary synthesis

## Abstract

After decades of debate about the existence of non‐genetic inheritance, the focus is now slowly shifting towards dissecting its underlying mechanisms. Here, we propose a new mechanism that, by integrating non‐genetic and genetic inheritance, may help build the long‐sought inclusive vision of evolution. After briefly reviewing the wealth of evidence documenting the existence and ubiquity of non‐genetic inheritance in a table, we review the categories of mechanisms of parent–offspring resemblance that underlie inheritance. We then review several lines of argument for the existence of interactions between non‐genetic and genetic components of inheritance, leading to a discussion of the contrasting timescales of action of non‐genetic and genetic inheritance. This raises the question of how the fidelity of the inheritance system can match the rate of environmental variation. This question is central to understanding the role of different inheritance systems in evolution. We then review and interpret evidence indicating the existence of shifts from inheritance systems with low to higher transmission fidelity. Based on results from different research fields we propose a conceptual hypothesis linking genetic and non‐genetic inheritance systems. According to this hypothesis, over the course of generations, shifts among information systems allow gradual matching between the rate of environmental change and the inheritance fidelity of the corresponding response. A striking conclusion from our review is that documented shifts between types of inherited non‐genetic information converge towards epigenetics (i.e. inclusively heritable molecular variation in gene expression without change in DNA sequence). We then interpret the well‐documented mutagenicity of epigenetic marks as potentially generating a final shift from epigenetic to genetic encoding. This sequence of shifts suggests the existence of a relay in inheritance systems from relatively labile ones to gradually more persistent modes of inheritance, a relay that could constitute a new mechanistic basis for the long‐proposed, but still poorly documented, hypothesis of genetic assimilation. A profound difference between the genocentric and the inclusive vision of heredity revealed by the genetic assimilation relay proposed here lies in the fact that a given form of inheritance can affect the rate of change of other inheritance systems. To explore the consequences of such inter‐connection among inheritance systems, we briefly review published theoretical models to build a model of genetic assimilation focusing on the shift in the engraving of environmentally induced phenotypic variation into the DNA sequence. According to this hypothesis, when environmental change remains stable over a sufficient number of generations, the relay among inheritance systems has the potential to generate a form of genetic assimilation. In this hypothesis, epigenetics appears as a hub by which non‐genetically inherited environmentally induced variation in traits can become genetically encoded over generations, in a form of epigenetically facilitated mutational assimilation. Finally, we illustrate some of the major implications of our hypothetical framework, concerning mutation randomness, the central dogma of molecular biology, concepts of inheritance and the curing of inherited disorders, as well as for the emergence of the inclusive evolutionary synthesis.

## I. INTRODUCTION

There is currently a heated debate about the necessity to modernize the modern synthesis of evolution (Pennisi, 2008; Bonduriansky, 2012; Danchin, 2013; Laland *et al*., 2014) into an ‘extended’ (Pigliucci & Muller, 2010; Laland *et al*., 2014; Wray *et al*., 2014) or ‘inclusive’ (Danchin *et al*., 2011; Danchin, 2013; Huneman & Whalsh, 2017) evolutionary synthesis. However, although usually not so formulated, this ongoing debate largely revolves around concepts of heredity and in particular the question of the existence and impact of non‐genetic inheritance.

**Figure 1:**
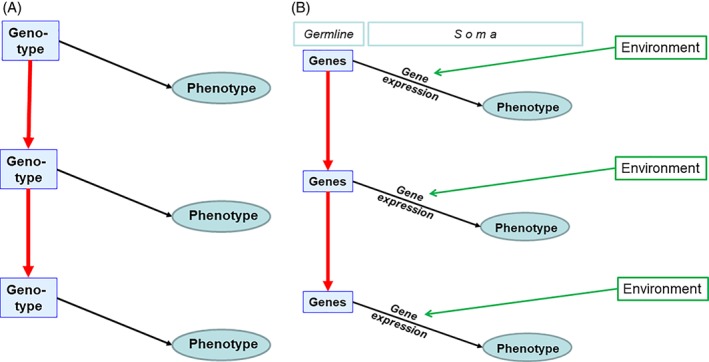
Inheritance according to the Modern synthesis. (A) Maynard Smith's (1965) vision describing two generations. (B) The canonical genocentric vision of inheritance according to the Modern Synthesis, where genes in the germline constitute the only significant information transfer across generations. Black arrows, development; plain red arrows, pathway of intergeneration information inheritance; green arrows, environmental effects.

Heredity, which results in patterns of parent–offspring resemblance, and the underlying mechanisms of inheritance, has long fascinated biologists and constitutes one of the foundations of biology as a scientific discipline. Lamarck (1809) is generally regarded as one of the first to propose a theory for the evolution of species. To him, famously, lineages change over time as a consequence of the use and disuse of organs (a phenomenon classically referred to as the inheritance of acquired characters). Darwin (1859) later proposed that, in addition to transmitting acquired characteristics, living beings also reproduce with variation, upon which natural selection can act. The laws of inheritance remained unknown to Darwin. Mendel (1866) independently established the fundamental laws of inheritance, but his work would have to be rediscovered at the beginning of the 20th century for his findings to become widely known. The question of the inheritance of acquired characters famously fuelled heated debate amongst biologists after Darwin. Weismann (1891) thought this mode of inheritance empirically was disproved and theoretically impossible. This view of inheritance is often summarized in a single diagram (Fig. 1) simplified in two steps from a diagram of Weismann (1891).

Baldwin (1896), Morgan (1896), and Osborn (1897) proposed that, in some cases, (non‐heritable) acquired characteristics at the level of the individual could enable a population to survive a new challenge until new hereditary variations appeared with the same physiological effect, and were selected for, thus mimicking a situation of inheritance of acquired characters. This model, however, remained controversial for decades after its proposal (see Simpson, 1953; Pocheville & Danchin, 2017).

The early population geneticists embraced similar views (Fisher, 1930; Mayr & Provine, 1998). To them, inheritance was purely genetic, sealed off from the environment. By the mid‐20th century, the discovery of the structure of DNA (Watson & Crick, 1953) and of the role of the linear sequence of nucleic acids in determining the structure of proteins, but not the other way around (Crick, 1958, 1970), reinforced the general opinion that inheritance concerns only the linear sequence of DNA, and that changes in this sequence are necessarily random. The idea of the inheritance of acquired characters, however, never totally faded away, and has steadily gained momentum over recent decades (Jablonka & Lamb, 1995, 2005, 2010; Morgan *et al*., 1999).

Over the past 60 years, the concept of heredity has been so inexorably reduced to the DNA sequence that it is now difficult to reopen it in order to incorporate epigenetics (Griffiths & Stotz, 2013; Lu & Bourrat, 2017). However, the material basis of heredity does not matter in evolutionary theory (Kronholm, 2017). The only important characteristics are that variation exists, affects fitness, and is inclusively heritable. Herein, the term ‘inclusive heritability’ means the proportion of phenotypic variation that is transmitted across generations, leading to parent–offspring resemblance, whatever the underlying mechanism of transmission, whether genetic or otherwise (Danchin & Wagner, 2010; Danchin *et al*., 2011). Nonetheless, progress in molecular biology at the turn of the third millennium – in particular with the development of high‐throughput sequencing – has produced a wealth of evidence that focusing only on the DNA sequence cannot fully explain the complexity of both parent–offspring resemblance and evolutionary processes.

The inclusive vision of inheritance that emerges from our review aims at unifying the inheritance systems as different facets of a single, pluralistic process of heredity (Jablonka & Lamb, 2005; Rando & Verstrepen, 2007; Pigliucci & Muller, 2010; Bonduriansky, 2012; Danchin, 2013; Noble, 2013; Kronholm, 2017). Unification, however, does not deny differences. As we develop herein, the transmission fidelities of diverse inheritance systems are crucial properties that enable them to encode adaptations to environmental characteristics with different turnover rates. It is thus crucial to identify them clearly as complementary and interacting facets of heredity in order to better unify them into an inclusive framework allowing us to keep track of their specific properties in terms of stability and transition between non‐genetic and genetic information.

### (1) Accruing evidence for non‐genetic inheritance

#### (a) *From accepting the reality of non‐genetic inheritance*…

The existence of non‐genetic inheritance has long been debated (among many reviews see Danchin *et al*., 2004, 2011; Mameli, 2004; Richards, 2006; Rando & Verstrepen, 2007; Franklin & Mansuy, 2010; Pigliucci & Muller, 2010; Bonduriansky, Crean & Day, 2011; Herman & Sultan, 2011; Chen, Yan & Duan, 2016b; Kronholm, 2017; Laland, 2017; Wang, Liu & Sun, 2017), but it is now widely accepted. Table 1 provides representative examples of the evidence supporting the existence and ubiquity of non‐genetic inheritance in organisms ranging from unicellular, to plants, to animals including humans.

**Table 1:** Selected examples that provide evidence for non‐genetic parent–offspring resemblance. The types of evidence include various terms used in the literature, although some of these overlap. Terms used in the right‐hand column correspond to the classification of the categories of mechanism of resemblance described in Section I.3: intergenerational effects (**IE**), multigenerational effects (**ME**) or transgenerational effects (**TrgE**); simultaneous exposure effects (**SEE**), germline‐independent transmission (**GIT**), or non‐genetic germline transmission (**NGGT**).

Type of evidence	Description of mechanisms	Resemblance mechanism
Epigenetic inheritance	We use the term ‘epigenetics’ to encompass all mechanisms linked to variation in gene expression and that are transmitted to the next generation along lineages of cells during development or along lineages of multicellular organisms. This variation in gene expression is associated with variation in three types of epigenetic marks that are either (*i*) fixed to the DNA molecule itself (methylation, acetylation), (*ii*) histone modifications (more than 100 such modifications have been documented), or (*iii*) involve small non‐coding RNAs that affect chromatin state, and thus control access to DNA (Halfmann & Lindquist, 2010). Many of the known examples of non‐genetic inheritance involve some sort of epigenetic inheritance. For example, in *Arabidopsis thaliana*, the phenotypic characterisation of epigenetic recombinant inbred lines (epiRIL) showed that flowering time and plant height have an epigenetic heritable basis in the absence of DNA sequence polymorphism and selection (Johannes *et al*., 2009). This variation was caused by variation in methylation across the genome that was stably inherited for at least eight generations	– Usually TrgE – NGGT
Inheritance of environmentally induced morphology in plants	Historically, one of the first striking examples of non‐genetic inheritance was documented in the toadflax (*Linearia vulgaris*) that exists in two heritable morphs, the most common one having flowers with a marked dorsoventral asymmetry, and a peloric form (originally described by Linnaeus) where flowers have a radial symmetry (Cubas, Vincent & Coen, 1999). This morphological polymorphism, which was one of the first natural morphological mutants to be described, actually results from heritable variation in the methylation of a single gene (*Lcyc*) known to affect flower asymmetry. In peloric forms, the gene is silenced by high methylation, and this methylated state can be stably transmitted over generations. This illustrates the extent to which epigenetic and genetic variation produce patterns of phenotypic change that are very difficult to distinguish. For ecological implications, see Herman & Sultan (2011) and Richards *et al*. (2017)	– IE, ME, TrgE – NGGT
Cultural inheritance	Following plant biology, the domain of cultural inheritance claimed that a genocentric vision of inheritance could not explain human evolution (Cavalli‐Sforza & Feldman, 1981, 1983; Boyd & Richerson, 1983 and 1985; Feldman & Cavalli‐Sforza, 1984) and cultural processes have been documented in various animals (insects: Alem *et al*., 2016; whales: Allen *et al*., 2013; great tits: Aplin *et al*., 2015; orangutans: van Schaik *et al*., 2003; chimpanzee: Whiten *et al*., 1999, 2011; Whiten, 2005, 2007, 2011; Whiten & Mesoudi, 2008; reviews in Avital & Jablonka, 2000; Danchin *et al*., 2004). Discrepancies between genetics and culture in whales (Whitehead, 1998; Rendell & Whitehead, 2001; Rosenbaum *et al*., 2002) and dolphins (Krutzen *et al*., 2005; Kopps *et al*., 2014) were documented, while cultural inheritance was claimed to be the only explanation for some human population genetic patterns (Heyer, Sibert & Austerlitz, 2005; review in Laland, Odling‐Smee & Myles, 2010). This suggests that cultural inheritance constitutes an important process of evolution that can drastically change the evolutionary fate of populations and species	– TrgE – GIT, possibly NGGT
Parental effects	The transmission of maternal behaviour in rats constitutes a model system to study the inheritance of maternal behaviour in humans (Beery & Francis, 2011). Female pups raised by normally caring dams show low levels of methylation of the promoter of genes coding for receptors to sexual hormones, while female pups regularly removed from their mother (i.e. with reduced maternal care), show high levels of methylation leading to the silencing of the corresponding genes (Francis *et al.,* 1999; Champagne, 2008; Champagne & Curley, 2009; Franklin & Mansuy, 2010). As adults, females that were raised by highly caring females express those receptor genes in their brain, making them sensitive to their own sexual hormones, and triggering a metabolic cascade that leads them to care for their own pups. By contrast, females that were artificially separated from their mother (mimicking low levels of care) do not express highly methylated sexual hormone receptor genes, affecting their sensitivity to their own sexual hormones, and leading them to neglect their own pups. This leads to persistent parent–offspring resemblance that is not based on variation the DNA sequence (for a review of such germline‐independent inheritance, see Bohacek & Mansuy, 2015)	–IE, ME, TrgE – Usually GIT
Ecological inheritance	Offspring often end up living in habitats that closely resemble that of their parents. This may be because they remain in their natal habitat, or because they disperse over short distances leading them to settle in the same habitat type, or more commonly because they were imprinted on their birth habitat, leading them to seek the same habitat type as adults (Teuschl, Taborsky & Taborsky, 1998). This generates parent–offspring resemblance in habitat choice, usually called ecological inheritance. While the initial establishment of the first ancestor may have been independent of its genotype, one consequence of ecological inheritance is that the lineage remains under the specific selective pressures of that habitat type as long as descendants manage to establish in their preferred habitat. This also occurs in humans where people born either in the country or cities tend to establish in their birth habitat	– TrgE – Probably GIT
Niche construction	Often confounded with ecological inheritance, niche construction results from the fact that living organisms modify their habitat, thus affecting selective pressures on subsequent generations. For instance, bacteria release acidic metabolic waste to their surroundings. Over many generations this means that the selective environment is likely to change gradually. In interaction with ecological inheritance, niche construction can considerably affect the evolutionary fate of populations (reviews in Odling‐Smee, Laland & Feldman, 2003; Odling‐Smee, 2010)	– TrgE – GIT
Inheritance of the fitness‐affecting effects of pollutants	In rats, endocrine‐disruptors commonly used in the wine industry strongly diminish the activity of the male germline. This effect is transmitted by male gametes at least until the F4 generation despite the fact that only the F0 females received the endocrine‐disruptor treatment (Anway *et al*., 2005; Skinner, Manikkam & Guerrero‐Bosagna, 2010). The disorder in the male germline and its transmission involve unusual methylation patterns in the testes. Similar multigenerational effects have been found with other contaminants (Skinner, Manikkam & Guerrero‐Bosagna, 2011)	– TrgE – NGGT
Environmental effects can persist for many generations in *C. elegans*	Studies in *Caenorhabditis elegans* underlined the role of non‐coding RNAs in the non‐genetic inheritance of environmentally induced phenotypes resulting from specific gene silencing. Such effects have been documented to persist for 3 (Greer *et al*., 2011), 14 (Klosin *et al*., 2017), 20 (Ashe *et al*., 2012), 25 (Devanapally, Ravikumar & Jose, 2015) and even 80 generations (for a review of epigenetic inheritance in *C. elegans* see Minkina & Hunter, 2018; see also Vastenhouw *et al*., 2006; Remy, 2010; review in Wang *et al*., 2017)	– TrgE – probably NGGT
Inheritance of traumatic exposure over several generations	F1 and F2 offspring of F0 mice subjected to odour fear‐conditioning before reproduction show an increased sensitivity to the usually ignored F0‐conditioned odour but not to other odours (Dias & Ressler, 2014; Szyf, 2014). The fear conditioning in F0 parents leads to hypomethylation in the gametes of the conditioned F0 parents of the specific gene involved in the reception of that odour. After *in vitro* fertilization of a gamete of a naive parent, this change in methylation pattern was maintained in the embryo and affected development such that the resulting adult F1 and F2 offspring showed fear of that specific odour and not others (review in Sharma, 2015; Szyf, 2015)	– TrgE – NGGT
Inheritance of acquired disorders	Parental environments can affect the phenotypes of offspring for several subsequent generations. In rodents, the triggering by high‐fat diet of the metabolic disorder underlying obesity and diabetes is transmitted to offspring even if the latter eat healthy food. This transmission occurs through the transmission of small non‐coding RNAs (sncRNAs) carried by sperm cells (Chen *et al*., 2016a; Zhang *et al*., 2018; review in Chen *et al*., 2016b). These RNAs are likely acquired by sperm cells during sperm transit in the epididymis (Sharma *et al*., 2016) in an intriguing form of soma to germen communication. This constitutes a promising animal model to study the transmission of acquired diabetes in humans, a major public health problem	– Probably TrgE – NGGT
Inheritance of microbiota	Dams in mammals transfer their skin and gut microbiota to their babies at birth and through lactation (Fellous, Duron & Rousset, 2011), which can lead to strong parent offspring resemblance. In humans, newborn babies are inoculated with their mother's microbiota at the time of birth in vaginally born infants and during lactation (review in Bonduriansky & Day, 2018; Makino *et al*., 2013). This could explain the striking mother–offspring similarity in their gut microbiota	– TrgE – GIT
Prions and other forms of molecular memory that are central in the transmission of disorders	Prions constitute an extreme case of epigenetic inheritance; instead of affecting access to the DNA as in all other epigenetic mechanisms, prions co‐opt the final step in the decoding of genetic information by affecting protein folding (Halfmann & Lindquist, 2010). Prions are proteins that can exist in several stable conformational states, which then template the configurational conversion of other molecules of the same protein. The resulting configurational change profoundly affects the properties of the protein, resulting in specific phenotypic changes. Prions thus constitute a form of long‐lasting molecular memory that, by being highly sensitive to environmental stress, could be involved in the acquisition and inheritance of new traits. They can thus be viewed as new molecules of heredity (Halfmann & Lindquist, 2010). Prions have also been found in wild strains of yeast (Halfmann *et al*., 2012), suggesting that they might play a significant role in non‐genetic inheritance of most living organisms	– Status, not documented – Probably NGGT

A key conclusion is that parent–offspring resemblance can result from diverse mechanisms documented in many domains of biology and that go well beyond DNA sequence transmission. These mechanisms include epigenetics [inclusively heritable (Danchin & Wagner, 2010) molecular variation in gene expression without change in DNA sequence, resulting from DNA methylation or histone modifications, and often mediated by small non‐coding RNAs (sncRNAs, i.e. RNA molecules that are not translated into a protein and that are less than 200 nt in size) (Morgan *et al*., 1999; Richards, 2006; Johannes, Colot & Jansen, 2008; Ashe *et al*., 2012; de Vanssay *et al*., 2012; Eichten & Borevitz, 2013; Cortijo *et al*., 2014; Kronholm, 2017; Wang *et al*., 2017; Nishikawa & Kinjo, 2018)], cultural and ecological inheritance (Danchin *et al*., 2004; Laland *et al*., 2010; Odling‐Smee, 2010; Fisher & Ridley, 2013), as well as parental effects (Francis *et al*., 1999; Jablonka & Raz, 2009; Bonduriansky *et al*., 2011; Danchin *et al*., 2011; Daxinger & Whitelaw, 2012; reviews in Mameli, 2004; Morgan *et al*., 1999; Richards, 2006; Sharma, 2015; Szyf, 2015). In its broadest meaning, non‐genetic inheritance also includes the vertical inheritance of symbionts (Fellous *et al*., 2011), as well as other modes of ‘inheritance’ such as prions (Manjrekar, 2017; Newby *et al*., 2017) and chaperone molecules (Halfmann & Lindquist, 2010; Lindquist, 2011; Halfmann *et al*., 2012; Saibil, 2013) that constitute other forms of molecular memory.

#### (b) *… to the study of its underlying mechanisms*

Today, the focus is slowly shifting towards the understanding of the mechanisms and evolutionary consequences of non‐genetic inheritance, which remains one of the major challenges of modern biology (Bonduriansky, 2012; Kappeler & Meaney, 2012; Danchin, 2013; Grossniklaus *et al*., 2013; Klironomos, Berg & Collins, 2013; Heard & Martienssen, 2014; Bohacek & Mansuy, 2015; Kronholm & Collins, 2015; Sharma, 2015; Szyf, 2015; Klosin & Lehner, 2016; Wang *et al*., 2017; Pujol *et al*., 2018). In this context, a crucial question is ‘whether there is a mechanism that could then fix these epigenetically driven phenotypic changes in the genetic sequence, thereby altering the course of evolution’ (Szyf, 2014, p. 4). This question which was first asked more than 20 years ago (Jablonka *et al.,* 1995), can now be tackled in the context of the wealth of evidence published since then and that we review in Section II.

### (2) Focus of this review

This review focuses on the molecular mechanisms of non‐genetic inheritance in order to unify them with genetic inheritance into a single inclusive evolutionary synthesis. During this process, a new framework emerges that we propose here as a working hypothesis. The main idea is that of the existence of a relay among inheritance systems from more labile to more stable forms of inherited information following a persistent environmental change. The idea of a relay among inheritance systems was suggested previously (review in Waddington, 1942, 1953, 1975; Jablonka & Lamb, 1995; West‐Eberhard, 2003; Pigliucci, Murren & Schlichting, 2006; Crispo, 2007), although none of these previous hypotheses proposed any molecular basis for the handovers along this relay. Here we propose that these handovers converge towards ‘epigenetic engraving’. Our hypothesis is that the effect of epigenetic marks on DNA mutability constitutes an excellent candidate for the last transition from epigenetic to genetic engraving of inherited information.

We first present a general classification of mechanisms of parent–offspring resemblance into three main categories depending on whether the resemblance has been shown only over one, two or more generations. We then revisit the relationships between non‐genetic and genetic inheritance, highlighting their contrasting transgenerational stability and timescales of action. We then review the evidence for potential shifts among inheritance systems. We propose that these shifts produce a relay among inheritance systems, eventually leading to genetic assimilation, thus favouring the matching of the transmission fidelity of the corresponding adaptation with the rate of environmental variation. Interestingly, we found evidence that many shifts (or ‘handovers’ in the relay) in inheritance systems seem to converge towards epigenetics. When this is the case, this epigenetic stage may generate a final handover towards genetic encoding. In this hypothesis, epigenetic marking constitutes a major hub linking non‐genetic germline inheritance with genetic inheritance. A profound difference between the genocentric and the inclusive vision of heredity revealed by the genetic assimilation relay proposed here lies in the fact that a given form of inheritance can affect the rate of change of other inheritance systems. We then use a simple theoretical model to investigate consequences of this relay in inheritance systems. We find that this form of epigenetically facilitated mutational assimilation (a term inspired by Jablonka & Lamb, 1995; see also Razeto‐Barry & Vecchi, 2017) may often accelerate considerably, and sometimes delay (Kronholm & Collins, 2015), the genetic encoding of adaptations. Finally, we discuss the implications and applications of this framework and conclude that we need to blend all inheritance systems in order to implement the long‐sought inclusive evolutionary synthesis.

### (3) Three broad categories of mechanisms of parent‐offspring resemblance

**Figure 2:**
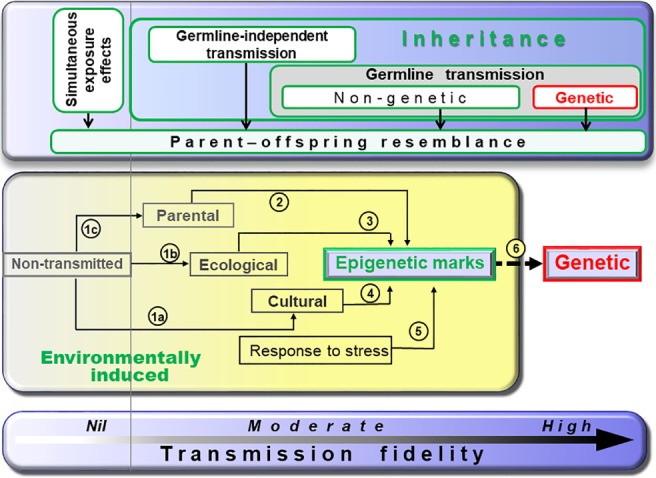
Epigenetics as a hub in the relay towards epigenetically facilitated mutational assimilation. The bottom panel provides a gradient of increasing transmission fidelity form left to right. The top panel shows categories of mechanisms of parent–offspring resemblance with their range of action represented by box width. The central panel shows pathways of genetic assimilation for which we review the empirical evidence herein. Any situation to the right from the non‐transmitted box (i.e. to the right of the faint vertical grey line) can lead to parent–offspring resemblance (i.e. heredity). Information encoding is thus relayed from relatively labile (left) to increasingly stable (right) inheritance systems, thus creating flows from the left to the right when environmental factors stabilize. Numbered arrows represent documented shifts in inheritance systems reviewed in the text. For example, arrow 6 illustrates that epigenetic marks are mutagenic so that the final engraving into genetics (i.e. genetic assimilation) unfolds over generations as a result of germline non‐genetic inheritance.

Mechanisms of parent–offspring resemblance encompass a variety of pathways that may or may not: (*i*) be genetic, (*ii*) involve the germline, or (*iii*) generate germline‐dependent transmission (Fig. 2).

In terms of non‐genetic inheritance, three categories are recognised on the basis of the number of generations during which resemblance has been experimentally demonstrated (Wang *et al*., 2017). Cases where resemblance is only demonstrated from F0 to F1 reveal *intergenerational effects*. Experiments in which resemblance continues from F1 to F2 show *multigenerational effects,* and when transmission has been demonstrated beyond F2 these are called *transgenerational effects* (Wang *et al*., 2017).

Another, largely (but not totally) overlapping, classification focuses on mechanisms of resemblance rather than patterns. It includes three categories of processes: simultaneous exposure effects, germline‐independent transmission and non‐genetic germline transmission (Anway *et al*., 2005; Skinner *et al*., 2010; Heard & Martienssen, 2014; Bohacek & Mansuy, 2015).

#### (a) *Simultaneous exposure effects*

In *simultaneous exposure effects* (Fig. 2), parent–offspring resemblance results from the simultaneous exposure of the pregnant female (hereafter exposed individuals are defined as the F0 generation) and its developing embryos (F1) (Skinner & Anway, 2005; Skinner *et al*., 2010; Heard & Martienssen, 2014) to a common environmental factor. The environmental factor may thus directly affect the pregnant F0 mother, its developing embryo (F1), as well as its already differentiated germline (future F2) leading to resemblance. Such cases do not necessarily involve transmission but rather the simultaneous exposure of several generations. Thus, when the treatment affected pregnant females, only traits that persist beyond F2 demonstrate transmission. When exposed individuals are males or non‐pregnant females, resemblance with F2 descendants is sufficient to demonstrate transmission of a character (Heard & Martienssen, 2014). Transmission may take one of two forms: germline‐independent transmission or non‐genetic germline transmission.

#### (b) *Germline‐independent transmission*

In non‐genetic *germline‐independent transmission* (Fig. 2), parent–offspring resemblance results from the fact that members of successive generations are exposed to the same environment for many generations, leading offspring to reconstruct the same traits *de novo* in each generation because they inherit the same environment (Bohacek & Mansuy, 2015). This occurs when offspring are behaviourally imprinted to their natal habitat early in life, and thus choose to live in the same type of habitats as adults. The lineage thus remains under the specific selective pressures of the preferred habitat for as long as individuals can establish in that preferred habitat. Germline‐independent transmission also includes cultural inheritance where behavioural patterns, as part of the offspring's environment, are acquired by social learning, potentially being transmitted over many generations. This is the case in rodents where early maternal care constitutes a major environmental component that generates offspring epigenetic patterns that lead them to reconstruct the same behaviour as their parents (Table 1, and e.g. Francis *et al*., 1999; Champagne *et al*., 2006). In mammals, the inheritance of microbiota during birth also belongs to this category. In germline‐independent transmission, offspring can be viewed as naturally inheriting components of the environment with the consequence that they resemble their parents over many generations.

#### (c) *Non‐genetic germline transmission*

In *non‐genetic germline transmission* (Fig. 2), the environment affects the germline in a way that persists across generations through various forms of molecular memory within the fertilizing gametes. The incomplete erasure of epigenetic marks in gametes was first demonstrated for the agouti phenotype in mice (Morgan *et al*., 1999), and since then further examples of non‐genetic germline transmission have been accruing (Table 1; Richards, 2006; Skinner *et al*., 2010; Danchin *et al*., 2011; Daxinger & Whitelaw, 2012; Castel & Martienssen, 2013; Grossniklaus *et al*., 2013; Heard & Martienssen, 2014; McCarrey, 2014; Sharma, 2015; Szyf, 2015; Wang *et al*., 2017).

Despite their profound mechanistic differences, all these categories lead to parent–offspring resemblance that can thus become the target of natural selection.

### (4) Shifts among inheritance systems generate a relay towards genetic assimilation

The existence of various categories of inheritance mechanisms raises the question of their roles in evolution (Manjrekar, 2017). A major difference between these mechanisms lies in their transgenerational stability (Fig. 2; Jablonka & Lamb, 1995; Klironomos *et al*., 2013). It was suggested that the more labile inheritance systems could convey information about environmental characteristics that change every few generations, while the more stable inheritance systems could encode information about longer‐lasting environmental characteristics (Jablonka & Lamb, 1995; Lachmann & Jablonka, 1996; Rando & Verstrepen, 2007; Klironomos *et al*., 2013; Herman *et al*., 2014). However, even inheritance systems with low transgenerational stability can profoundly affect evolutionary dynamics in the long term (Jablonka & Lamb, 1995; Klironomos *et al*., 2013; Kronholm & Collins, 2015), and it has long been known that adaptive processes occurring at the developmental timescale can be effective in catalysing evolution (Hinton & Nowlan, 1987; West‐Eberhard, 2003; Laland *et al*., 2010).

Matching the pace of environmental change to transmission fidelity should be evolutionarily advantageous (Herman *et al*., 2014). This suggests that a mechanism allowing phenotypic encoding to shift between inheritance systems towards more stable inheritance under low rates of environmental change would be positively selected. Historically, such a shift corresponds to the model of genetic assimilation that depicts situations where some phenotypic variation initially caused by environmental change eventually becomes encoded into the DNA sequence if the initial environmental change persists for a number of generations (Waddington, 1942, 1953; West‐Eberhard, 2003; Pigliucci *et al*., 2006). Although genetic assimilation has classically been understood in the Modern Synthesis framework as produced by the selection of pre‐existing hidden or standing genetic variation in the population (Crispo, 2007), here we review evidence for how environmental change can make a novel adaptation heritable by genuinely inducing shifts in information encoding that occur during an organism's lifetime. Thus, when a previously unstable environmental factor stabilizes, the information enabling an adaptive response to that factor can shift from relatively unstable to increasingly stable inheritance systems rather like passing a baton from the first (labile) to the last (stable) runner in a relay race (Fig. 2).

The term transgenerational plasticity is usually used to depict cases where a plastic trait is transmitted to subsequent generations. It is a mechanism that is tightly linked to that of ‘genetic accommodation’ (see Crispo, 2007; a term introduced by West‐Eberhard, 2003), by which new environmentally induced phenotypes become inclusively heritable. Genetic accommodation implies a change in the way the trait is encoded, without necessarily implying change in the DNA sequence, as is thought to be the case in genetic assimilation.

Interestingly, even Waddington's (1942, 1953, 1959) seminal experiments could represent genetic accommodation rather than genetic assimilation as usually understood. In particular, his results might have been mediated by the effect of chaperone molecules like heat shock proteins such as Hsp90 (Rutherford & Lindquist, 1998; Nishikawa & Kinjo, 2018). Furthermore, duplication of Waddington's experiments found similar results, even when using an inbred *Drosophila melanogaster* line and in the absence of selection (Ho *et al*., 1983), suggesting that the classical interpretation that genetic assimilation is produced by the selection of cryptic genetic variation is not sufficient. Another replicate of Waddington's experiments provided statistical evidence that selection affected some inherited variation closely linked to the *Ubx* gene, but did not provide evidence that the concerned variation was in terms of DNA sequence (Gibson & Hogness, 1996).

There are many instances in which a heritable phenotypic change first attributed to a genetic change (i.e. a change in the DNA sequence) was later proved to be due to a functionally efficient heritable change in epigenetic marks. The most famous example is that of the toadflax (*Linearia vulgaris*) that exists in two highly heritable morphs, the most common having flowers with a marked dorsoventral asymmetry, and a peloric form (originally described by Linnaeus) where flowers have radial symmetry (Cubas *et al*., 1999). This morphological polymorphism, which constituted one of the first natural morphological mutants ever described, is in fact not a genetic mutation, but rather the result of heritable changes in methylation at a single gene (*Lcyc*) affecting flower asymmetry, which appears to be silenced in the peloric morph. This shows the extent to which epigenetic and genetic variation produce patterns of phenotypic change that are very difficult to distinguish. Many other examples are provided in Table 1.

## II. ALL ROADS LEAD TO GENES, *VIA* THE EPIGENETIC HUB

We now review evidence suggesting that environmentally induced heritable effects converge towards epigenetics, which can then facilitate the genetic encoding of inherited information owing to the mutagenicity of epigenetic marks. A striking outcome of this review is that epigenetic transmission provides a general molecular mechanism for parental effects. Thus, in being epigenetically mediated, environmental effects have the potential to bridge the short‐term timescale of adaptive plastic responses with the much longer timescale of adaptive evolutionary responses.

### (1) From non‐transmitted to transmitted information

The relay starts when previously non‐transmitted variation becomes transmitted. For example, behavioural innovations are reinvented regularly until social learning eventually triggers transgenerational stability (arrow 1a, Fig. 2). Similarly, initially non‐transmitted characteristics may become inherited ecologically as with the beavers' dam (arrow 1b, Fig. 2) or parentally as with transferred immunity (arrow 1c, Fig. 2).

### (2) From parental effects to heritable epigenetic marks

The second step is when parental effects lead to heritable epigenetic marks (arrow 2, Fig. 2). An example is when variation in maternal care is maternally transmitted to daughters over generations (Denenberg & Whimbey, 1963; Francis *et al*., 1999; Weaver *et al*., 2004; Champagne *et al*., 2006; Beery & Francis, 2011; Table 1). Variation in maternal care triggers the differential epigenetic marking of daughters' genes coding for receptors to sexual hormones (Champagne, 2008). As a result, variation in daughters' brain sensitivity to their own sexual hormones is induced, which reconstructs the same level of maternal care in the adult daughters, leading to persistent variation in maternal care among lineages (Francis *et al*., 1999; Champagne *et al*., 2006; Beery & Francis, 2011). This constitutes a classic case of *germline‐independent transmission* in which epigenetically induced maternal behaviour becomes the environmental cause of the reconstruction of similar epigenetic marks in their developing daughters, and continues over many generations, leading to persistent mother–daughter resemblance in maternal care.

More generally, parental capacity to modulate their offspring's epigenetic marks constitutes an ideal candidate inheritance mechanism for germline‐independent inheritance (Kappeler & Meaney, 2012), and rodent studies have been useful in identifying risk‐factors relevant to humans (Beery & Francis, 2011).

### (3) From ecological effects to heritable epigenetic marks

Another fascinating pathway links ecology to heritable epigenetic marks (arrow 3, Fig. 2). For instance, stem elongation in response to shade is common in plants (Schmitt, 1997). In *Stellaria longipes*, this non‐genetic change has been linked to a lower level of DNA methylation (Tatra *et al*., 2000). Furthermore, in *Campanulastrum americanum*, a forest plant inhabiting the understorey or open areas, experiments showed that seeds planted in the same light environment as their maternal plant had 3.4 times higher fitness than sibling seeds in the alternative light environment (Galloway & Etterson, 2007). Such ecologically transmitted non‐genetic priming of seeds will be adaptive as most seeds disperse over very short distances, and will therefore germinate in the same light environment as their parent. Thus, some sort of heritable information in these seeds primes them for the habitat in which they will germinate (Galloway & Etterson, 2007).

Such studies raise the question of the molecular pathways by which such environmental features affect development (Szyf, 2014, 2015; Richards *et al*., 2017; Wang *et al*., 2017). A suite of studies in *Caenorhabditis elegans* (Ashe *et al*., 2012) underline the major role of various types of non‐coding RNAs in the inheritance of acquired traits with transgenerational effects persisting over more than 14 (Klosin *et al*., 2017), 25 (Devanapally *et al*., 2015) and even 80 generations (Vastenhouw *et al*., 2006; Minkina & Hunter, 2018; review in Wang *et al*., 2017).

One of the most striking examples of inheritance of environmentally triggered responses links parental environment to the phenotype of their descendants in mice (Sharma *et al*., 2016) where metabolic disorders associated with obesity and diabetes can result from a paternal high‐fat diet (HFD) (Chen *et al*., 2016a). Male offspring of HFD males mated to normal females develop the two components of the disorder (glucose intolerance and insulin resistance) even if fed a healthy diet. Furthermore, injecting a single sperm head from a HFD male into an oocyte from a female that did not have the disorder induces the resulting male offspring to develop the full disorder as if a HFD male had sired them. This demonstrates that the sperm head contains all the information to develop the disorder. Surprisingly, part of the inherited information for the development of the disorder seems to be contained in a small fraction of sperm cell RNA extracts, because injecting a specific fraction of RNA extracts [transfer RNA‐derived small RNAs (tsRNAs) of 30–40 nt in size] from sperm of HFD males leads to the development in the resulting offspring of the glucose‐intolerance part of the disorder, but not the insulin‐resistance component (Chen *et al*., 2016a). A similar phenomenon was described in *D. melanogaster* (Ost *et al*., 2014). In mice, these sperm cell tsRNAs are incorporated into sperm cells while they transit through the epididymis (Sharma *et al*., 2016). Furthermore, RNA‐filled seminal exosomes exist in several species, including humans (Vojtech *et al*., 2014). The lumen of the epididymis duct contains many of these RNA‐filled micro‐exosomes. By fusing with sperm cells, these vesicles are strongly suspected to incorporate their RNA content into the sperm cells (Sharma *et al*., 2016) in a surprising form of soma‐to‐germen‐communication with transgenerational effects.

It is widely accepted that the environment can affect development and thus phenotype. However, our knowledge about the underlying mechanisms and their interactions remains limited. The studies described in this section all suggest epigenetic pathways by which environmental characteristics can affect development. A surprising result is that the underlying epigenetic changes (including DNA methylation, histone modifications and sncRNAs) often can be transferred across generations (Cubas *et al*., 1999; Vastenhouw *et al*., 2006; for a review see Wang *et al*., 2017). These results imply that some RNA fractions contain subtle information allowing the reconstruction of the same phenotype for many generations, resulting in *non‐genetic germline transmission*.

### (4) From social heredity to heritable epigenetic marks

Epigenetics is also involved in long‐term memory systems, which can link cultural transmission to its potential epigenetic bases (arrow 4, Fig. 2) (reviews in Fischer, 2014; Tuesta & Zhang, 2014). Studies of memory consolidation and transmission across generations show that all forms of epigenetic marks can participate in memory formation, often with heritable effects (Fischer, 2014; Tuesta & Zhang, 2014). For instance, fear conditioning jointly leads to the hyper‐methylation of a memory‐suppressor gene and the hypo‐methylation of a memory‐promoting gene in rats (Miller & Sweatt, 2007). In mice, chronic separation from the mother induces depressive‐like behaviour in F0 separated animals when adult. Furthermore, although raised in normal conditions, F1, F2 and F3 offspring of the F0 males display most of the behavioural alterations of the F0 adult males (Franklin *et al*., 2014). Furthermore, maternal separation was shown to alter the DNA methylation profiles of the promoters of genes associated with depression and emotions in the germline and brain of separated males (Franklin *et al*., 2014). Altogether, such results show that social heredity may involve epigenetics in the generation of both *germline‐independent transmission* and *non‐genetic germline transmission* of behaviour.

### (5) From stress response to germline heritable epigenetic marks

More direct bridges between environmental and inherited epigenetic changes are known. There are fascinating examples of a single environmental stress directly leading to new phenotypes that persist for at least several generations through *epigenetic germline transmission* well after the disappearance of the environmental stress (arrow 5, Fig. 2).

A classic example in rats concerns the transgenerational action of hormone disruptors commonly used in the wine industry, through modifications of the male germline (Anway *et al*., 2005). Nearly all F1–F4 male descendants of F0 pregnant females treated with such chemicals showed strongly decreased fertility concomitant with unusual methylation patterns in the testes. The expression of over 400 genes in F3 appeared affected by the treatment three generations earlier (Guerrero‐Bosagna *et al*., 2013). Furthermore, preference tests showed that F3 females (but not males) of treated F0 pregnant mothers (as well as females with no history of exposure) preferred males whose progenitors were not exposed to endocrine disruptors over males whose progenitors were exposed three generations earlier (Crews *et al*., 2007). This suggests that such effects can affect the fitness of descendants and thus act as a focus for natural selection (Crews *et al*., 2007). Decreased fertility was transmitted over at least four generations by male but not female gametes despite the fact that only the F0 female received the hormone disruptor (Anway *et al*., 2005) or other contaminants (Skinner *et al*., 2011).

Mice provide another fascinating example of environmentally acquired traits that are directly epigenetically inherited over several generations (Dias & Ressler, 2014). Experiments demonstrated that parent mice of both sexes conditioned by the association between a benign odour and a mild electric shock hypomethylate the corresponding olfactory receptor gene in their gametes. Furthermore, after *in vitro* fertilizations of unexposed female ova by sperm of exposed males (or *vice versa*), this methylation pattern was transmitted to unexposed F1 and F2 offspring that then feared the same odour (but not a different odour) when first exposed to it.

These results raise puzzling questions on how an environmental trigger could affect epigenetic marks on specific genes in the germ cells (Sharma, 2015; Szyf, 2015). One possible answer might involve double‐stranded RNAs (dsRNA) from somatic cells. In *C. elegans*, neurons produce and release dsRNA that can reach the germline causing transgenerational silencing that lasts for at least 25 generations (Devanapally *et al*., 2015). Similarly in mice, experimental manipulation of sperm RNA content generated father–offspring resemblance in a chronic stress phenotype (Rodgers *et al*., 2015) and in diabetes (Chen *et al*., 2016a). Further evidence for a major role of maternal RNA in intergenerational transmission of induced phenotypes (Ost *et al*., 2014) and of transgenerational inheritance even over 24 generations was documented in *D. melanogaster* (Stern *et al*., 2012, 2014). Thus, RNA‐mediated inheritance emerges as a major molecular pathway of *non‐genetic germline transmission* (Daxinger & Whitelaw, 2012; Chen *et al*., 2016a; Wang *et al*., 2017).

These examples show not only that the environment can affect phenotypes, but also that the resulting phenotypic change can be epigenetically transmitted through the germline for up to at least 80 generations (Vastenhouw *et al*., 2006; review in Wang *et al*., 2017). It should be noted that most published estimates of transgenerational persistence probably constitute minimal values because most studies stop before the disappearance of the environmental effect. Such *non‐genetic germline transmission* questions the concept that the germline is protected from environmental effects. Clearly, germ cells are not sealed off from environmental influences, but environmental effects can trigger sophisticated pathways in somatic cells that directly target germ cells (Devanapally *et al*., 2015; review in Sharma, 2015; Szyf, 2015; Rey *et al*., 2016; Wang *et al*., 2017).

### (6) Epigenetic marks are mutagens

#### (a) *Epigenetic marks foster point mutations*

Perhaps the most direct pathway in the genetic assimilation relay from non‐genetic to genetic inheritance (arrow 6, Fig. 2) involves epigenetically mediated mutations due to the mutagenicity of epigenetic marks (Makova & Hardison, 2015; Rey *et al*., 2016). Many epigenetic features (including histone, covalent modifications of histone tails and nucleotides, genomic landscape features, and small RNAs) can affect mutation rates (Jablonka & Lamb, 1995, 2005; Glastad *et al*., 2015; review in Sawan *et al*., 2008; Schuster‐Boeckler & Lehner, 2012 Makova & Hardison, 2015; Polak *et al*., 2015).

Links between epigenetic marks and mutation rates are best documented for DNA methylation patterns, which were first demonstrated to be mutagenic in *E. coli* in 1978 (Coulondre *et al*., 1978; Duncan & Miller, 1980). Overall at the molecular level, as the rate of deamination of 5‐methylcytosine into thymine is about 3.5 times higher than that of unmethylated cytosine into uracil (Jones *et al*., 1992), and as mismatched uracils are excised up to 6000 times more efficiently than mismatched thymines (Schmutte *et al*., 1995), the mutation rate of 5‐methylcytosine appears about 20000 times higher than that of unmethylated cytosines (Gorelick, 2003). As a consequence, methylated cytosines are suspected to cause 30–40% of germline point mutations in humans (Jones *et al*., 1992). This very large difference in genomic stability results from the cumulative effects of natural cytosine and 5‐methylcytosine deamination, plus the differential efficiency of mismatch repair and maintenance methylation, plus natural tautomeric shift processes (Gorelick, 2003). It has also been suggested that this difference in mutability may result from the joint action of various types of covarying epigenetic marks or their interactions (review in Makova & Hardison, 2015).

Less is known, however, about how such point mutations translate into point mutation rates at the population level. The mutagenicity of DNA methylation is best documented in primate populations including humans where methylcytosine is viewed as a potent mutagen (Sawan *et al*., 2008; Schuster‐Boeckler & Lehner, 2012). For instance, in several population studies, cytosine methylation at a CpG dinucleotide increases the probability of a C to T change (or the corresponding G to A) by a factor of 12–42 (Gonzalgo & Jones, 1997). In primates including humans, this mutagenicity increases mutation rates by an average factor of 15 (Elango *et al*., 2008). Similarly, the identification of more than 12 million biallellic and uniquely mapped single nucleotide polymorphisms over the whole human genome has provided support to the idea that the mutation rate in methylated CpGs is greater than in unmethylated CpGs, with estimated mutation rates being up to 20 times higher in some methylated parts of the genome (calculated from Xia, Han & Zhao, 2012). Furthermore, in humans, chromatin accessibility and modifications in conjunction with replication timing explained 86% of the variance in mutation rates along cancer genomes (Polak *et al*., 2015).

A striking result on methylcytosine point mutagenicity is thus that while the molecular stability between cytosine and methylcytosine differs by a factor of 20000 (Gorelick, 2003), at the population, level observed mutagenicity ranges only between 10 and 50 times (Gonzalgo & Jones, 1997). This difference may indicate that most mutations are deleterious with around 1 in 400 being viable enough for their bearers to survive until sampling. These considerations indicate that epigenetically induced point mutations probably play a role in a vastly larger number of new genetic variants than we can actually detect.

#### (b) *Genomic landscape features affect regional changes in DNA sequence*

At the larger scale of genomic regions (i.e. at the scale of the genomic landscape), various studies suggest that the statistical link between epigenetic marks or chromatin state and mutation is very general (Haines, Rodenhiser & Ainsworth, 2001; Sawan *et al*., 2008) and also affects germline and stem cells (reviews: Daxinger & Whitelaw, 2012; Schuster‐Boeckler & Lehner, 2012; Xia *et al*., 2012; Makova & Hardison, 2015; Polak *et al*., 2015). For instance, at the regional scale, epigenetic change has long been suspected to cause the early stages of tumour genesis, as regional epigenomic changes, and particularly DNA methylation, often precede cancers (Gonzalgo & Jones, 1997; Plass & Soloway, 2002; Sawan *et al*., 2008; Makova & Hardison, 2015). More generally, various genomic landscape features are suspected to act in synergy to explain variation in mutation rate (review in Makova & Hardison, 2015). Thus, mutations involved in tumour genesis may be considered more as a consequence of disrupted epigenetic states than the initial cause of cancer (Jones *et al*., 1992).

More generally, differential rates of mutation in methylated DNA regions predict an association between differentially methylated regions (DMRs) and local DNA‐sequence variation. This association was documented in a large epigenome‐wide association study in *Arabidopsis thaliana* involving more than 150 wild individuals presenting phenotypic variation (Eichten & Borevitz, 2013; Schmitz *et al*., 2013). More than 30% of the DMRs were also regions of higher DNA‐sequence variation. However, most information at this level results from correlations between chromatin landscape features and mutation rates, and the causality of this association remains to be fully explored (Ehrlich & Wang, 1981; Huttley, 2004; Schuster‐Boeckler & Lehner, 2012; Makova & Hardison, 2015; Polak *et al*., 2015).

While the link between methylation and mutation at both point and larger scales is well documented, the consequences that we suggest in terms of genetic assimilation appear, however, not to be currently supported by empirical data. For instance, a pioneer experimental evolution over 200 generations in *Chlamydomonas reinhardtii* found that ‘differences in methylation patterns were not associated with nearby genetic mutations’ (Kronholm *et al*., 2017, p. 2286). Such findings do not corroborate our proposed hypothesis. However, the lack of replication of such experimental evolution studies specifically designed to investigate the role of epigenetic inheritance makes it difficult to extract general principles. Even this impressive experiment might not be sufficient to detect the effect of the mutagenicity of epigenetic marks. If natural mutations rates are below 10^–7^ (as frequently reported, examples in Klironomos *et al*., 2013), epigenetically facilitated mutations would need around 10^3^ generations to occur at specific loci with a mutagenicity of epigenetic marks of 10^4^. This raises the question of the timescale of such experimental detection studies. Furthermore, a recent theoretical paper concluded that in a large population of ∼10^5^ individuals, the time for a single favourable mutation to reach a significant (and thus detectable) fraction of the population may take between 30 and 140 generations (Denman, 2017). Alternatively, epigenetically facilitated mutations can emerge more rapidly in large populations such as in microbes. The sequencing of multiple individuals would in this case considerably increase our capacity to detect epigenetically facilitated mutations after 200 generations. Finally, as the epigenetically facilitated point mutations erase the corresponding methylations, one would also need to sequence and episequence individuals regularly along the lineage to be able to show that the epigenetic change pre‐dated the mutation. Thus, the detection of epigenetically facilitated mutations will need very specific experiments.

The long timescale of epigenetically facilitated mutational assimilation is probably adaptive because a faster engraving of adaptive responses into genes would be too rapid in view of the irreversibility of the genetic encoding, which probably only becomes adaptive after the environmental trigger has stabilized for a sufficient number of generations to demonstrate very high stability.

#### (c) *Epigenetic marks and transposable elements foster large‐scale genetic change*

Another straightforward pathway linking environmentally induced epigenetic modifications to genetic engraving lies in the tight regulation of transposable elements (TEs) by epigenetic marks (Reinders *et al*., 2009; Zeh, Zeh & Ishida, 2009). TEs can copy and/or transpose themselves over the genome (Wicker *et al*., 2007). Their abundance is heterogeneous across the tree of life, sometimes representing up to 64% of the genome (Sotero‐Caio *et al*., 2017). For instance, half of the mammalian genome (around 45% in humans) derives from transposable elements, most of which are inactive (Cordaux & Batzer, 2009; Sotero‐Caio *et al*., 2017). Their transposition rate varies greatly among organisms and tissues, but is higher in germinal cells and generally strongly repressed in differentiated somatic cells (Haig, 2016; Tiwari *et al*., 2017). Although usually inactivated by epigenetic marks, TEs can be reactivated by environmentally induced modifications of these repressive epigenetic marks (Zeh *et al*., 2009; Fedoroff, 2012). When activated, TEs are important sources of genetic variation and genomic reorganization (Feschotte, 2008; Chenais *et al*., 2012; Stuart *et al*., 2017). Moreover, some TEs intrinsically include regulatory elements (e.g. enhancers) that can modify gene expression in the neighbourhood of their new insertion sites (Chuong, Elde & Feschotte, 2017). Furthermore, in both *Drosophila* and zebrafish, diverse classes of retrotransposons were recently shown to act ‘as molecular stowaways to gain passage from their site of production … to the oocyte germ plasm’ (Tiwari *et al*., 2017, p. 3013), leading them ‘to invade rudimentary components of germ cells that eventually form “grandchildren”…’ (Tiwari *et al*., 2017, p. 3013). In other words, TEs can be produced in somatic cells and rapidly migrate to germ cells and thus become inclusively heritable.

Although *de novo* insertions of TEs can be deleterious (they are associated with at least 96% of genetic diseases in humans, including cancers; Burns, 2017; Hancks & Kazazian, 2012), accruing evidence indicates that they may foster the emergence of adaptive phenotypes and/or regulatory pathways (Rebollo, Romanish & Mager, 2012; Miousse *et al*., 2015; Rey *et al*., 2016). TEs also are a major source of within‐population genetic variation (Stuart *et al*., 2017). TEs are thus powerful facilitators of genomic evolution (Oliver & Greene, 2009) and have played a crucial role in major evolutionary transitions (Agrawal, Eastman & Schatz, 1998; Daboussi & Capy, 2003; Gonzalez *et al*., 2008; Lisch, 2013), including the evolution of hominid brain size, immune defence, reproduction and development (Britten, 2010; Oliver & Greene, 2011; Koonin & Krupovic, 2015). Recent studies also highlight the potential role of TEs in shaping adaptive responses of organisms at contemporary scales (Casacuberta & Gonzalez, 2013; Rey *et al*., 2016). This is illustrated by their contribution to the rapid emergence of phenotypic variants resistant to man‐made insecticides in wild invertebrate populations (Rostant, Wedell & Hosken, 2012).

In this environment–epigenetic–TE triptych, epigenetic marks constitute key elements able to ‘translate’ environmental cues perceived by the organism into large‐scale genomic change. Under stressful conditions, this complex molecular engine promotes the emergence of epigenetically driven phenotypic and genomic variation upon which selection may act, while stabilizing phenotypes and genomes under the usual constant environmental conditions (Rey *et al*., 2016). As such, epigenetic components act as conductors fine‐tuning an organism's evolvability in response to changing selective pressures (Rando & Verstrepen, 2007; Rey *et al*., 2016).

Finally, it is worth stressing that the insertion sites of TEs are usually non‐random because some specific nucleotidic sequences, chromatin and nuclear contexts may partly guide the location of their *de novo* integration (Sultana *et al*., 2017). This implies that environmentally induced epigenetic modifications may promote and guide the insertion of TEs into specifically targeted genomic regions. Together these fascinating properties suggest that the environment not only acts as a selective filter on stochastically emerging variants but may also promote the emergence of non‐random epigenetic and genomic variants in a timely and targeted fashion according to the specific ongoing selective pressures. The tight link between epigenetic marks and TEs thus may greatly facilitate the molecular relay from epigenetically encoded to genetically encoded information, and may thus be an inclusive part of the epigenetically facilitated mutational assimilation we discuss here.

### (7) Epigenetics as a hub towards genetic assimilation

The wealth of evidence from different disciplines reviewed in this section provides support for the theoretical concept that epigenetic germline transmission can act as a hub towards genetic assimilation. According to this idea, the germline epigenetic state is expected to covary with environmental variation whatever its rate of variation, eventually affecting the DNA sequence over the course of multiple generations. Thus, after a significant environmental shift from a stable environmental state to novel but stable environmental conditions, traits may first become inclusively heritable (i.e. variation is transmitted non‐genetically), and eventually become genetically encoded, provided that the new environment remains stable for sufficient time for this multi‐generation mechanism to induce mutations that can then be selected. It thus corresponds to a ‘*mutational assimilation*’ (a term introduced by Jablonka & Lamb, 1995; see also Razeto‐Barry & Vecchi, 2017) in which mutations are facilitated by epigenetics. We thus call it ‘epigenetically facilitated mutational assimilation’.

Intuitively, the proposed mechanism linking epigenetic and genetic changes has the potential to accelerate genetic evolution. Below, we present a model studying the extent to which this form of genetic assimilation can accelerate the genetic encoding of acquired heritable adaptations.

## III. A MODEL OF EPIGENETICALLY FACILITATED MUTATIONAL ASSIMILATION

We now outline a model to explore the possible evolutionary consequences of epigenetically facilitated transfer of information between the environment and the epigenetic and genetic materials. The model simulates the evolution of populations experiencing a sudden environmental change that shifts an adaptive peak. We compare populations with specific strategies of hereditary transmission to assess how rapidly they can reach the new fitness peak. For that goal, as in previous studies (Herman *et al*., 2014), we present results of simulations in which we fix the selection pressure generated by environmental change for an indefinite number of generations to analyse which strategy is the most efficient in reaching the new fitness peak. However, it is important to note that simulating a fluctuating evolutionary challenge would yield similar results.

### (1) Model specification

The model is based on Klironomos *et al*. (2013), with the addition of other putative mechanisms of genetic assimilation (Jablonka & Lamb, 1995) and exploring other strategies (see online Supporting Information Table S1 for a comparison with previous models exploring genetic and mutational assimilation). Individuals are modelled as a couple of one genetic variable and one epigenetic variable (the latter called an ‘epigene’). Each variable is represented by a sequence of *k* and l bits, respectively; each sequence mutates with a determined rate (μ_gene_ < μ_epigene_; all parameters are described in Table S2, Supporting Information). This does not necessarily mean that the corresponding biological variables are genuine sequences, but rather that we chose to represent them as binary objects for convenience. Individual fitness is determined by the value of both the genetic and the epigenetic variables. We consider here the case where genetic and epigenetic materials can lead to similar effects (for instance, a gene can be silenced for genetic or epigenetic reasons). Thus *w*_individual_ = max[*w*_gene_, *w*_epigene_, where *w* is fitness]. Following the model of Klironomos *et al*. (2013), the adaptive landscape is single‐peaked for each variable, and flat otherwise. For simplicity, we consider that the genetic and epigenetic adaptive landscapes are identical (i.e. *w*_gene peak_ = *w*_epigene peak_ and *w*_gene off‐peak_ = *w*_epigene off‐peak_), except when epigenes are costly to maintain (*w*_costly epigene peak_ < *w*_gene peak_). The population is limited to a fixed carrying capacity.

### (2) Strategies investigated

**Figure 3:**
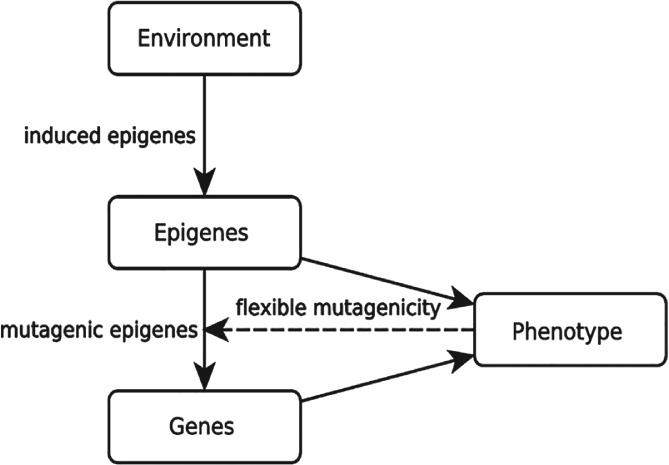
General diagram of the model.

We explore a series of strategies of inheritance mechanisms (Fig. 3). Importantly, the model is an abstract exploration of the biological possibilities, and the strategies do not necessarily correspond to mechanisms realized in the biological world. To keep the exposition simple, we also ignore maladaptive strategies or severe adaptive landscapes resulting in population extinction.

The simplest strategy is *genes‐only*: individuals consist of only one genetic sequence, mutating with rate μ_gene_. A similar strategy is ‘*epigenes‐only*’ (one epigenetic sequence, mutating with rate μ_epigene_). This amounts to the gene‐only strategy, with a higher mutation rate. All other strategies use both genes and epigenes. With the strategy *genes‐and‐epigenes*, genes and epigenes mutate independently (according to their respective mutation rates). These strategies correspond to the Klironomos *et al*. (2013) model. With the strategy *mutagenic‐epigenes*, epigenes increase the mutation rate for the whole gene (by a factor of 10^2^, proportionally to the number of epigenetic bits that are equal to 1). This strategy is similar to that of Jablonka & Lamb (1995). This simulates the mutagenic effect of some epigenetic marks as documented above. With the strategy *inducible‐mutagenic‐epigenes*, epigenes increase the genetic mutation rate as above, and are themselves induced towards fitness when they mutate (each mutated epigenetic bit turns into a 1, we explored a range of rates of induction towards fitness, reaching similar results). This simulates a situation where regulatory patterns, or maternal effects, are adaptively induced by the environment. In this strategy and the following ones, epigenetic marks generate new variation that is then open to selection in the model.

We also explore the case where maintaining an epigenetic system is costly (e.g. due to costs of protein synthesis, or lags in reaction to the environment at the intra‐generation timescale). The strategy *costly‐inducible‐mutagenic‐epigenes* is identical to the *inducible‐mutagenic‐epigenes* one, except that the fitness peak for epigenes is inferior to the fitness peak for genes.

Last, we consider a situation of adaptive plasticity: the induction of epigenes is context‐dependent. More precisely, epigenes are induced towards fitness when and only when the individual is not fit, and epigenes mutate randomly otherwise (the strategy is called *flexible‐inducible‐mutagenic‐epigenes*). This strategy can be thought of as simulating a situation of mutational assimilation (Jablonka & Lamb, 1995), where the genetic variable is defective, off‐peak, and where the cellular machinery up‐regulates gene expression until a certain physiological result is obtained. The physiological result can be obtained either by up‐regulation (epigene on peak) or by mutating the gene (gene on peak). Up‐regulation is assumed to be itself mutagenic (e.g. Wright, 2000). In this case, mutagenicity can be thought of as an exaptation of plasticity (Pocheville & Danchin, 2017).

### (3) Simulation run

Only one strategy of hereditary transmission is tested at a time. The starting population is monomorphic, offpeak (all bits set to 0). The dynamics consists of a succession of generations where: (*i*) individuals reproduce (according to their fitness), individuals in excess, if any, are randomly removed; (*ii*) for each remaining individual, the genetic and epigenetic sequences undergo possible mutations (according to their respective rates). Each simulation is run for 10^6^ generations.

At each generation, the fitness of the population (before truncation) is recorded, and the geometric average of the fitness since the beginning of the simulation is computed. The geometric average at a given time indicates which strategy would win (i.e. be more numerous) at this time (were the strategies actually competing), thus indicating the timescales at which the strategy is adaptive in comparison to the others.

### (4) Results

**Figure 4:**
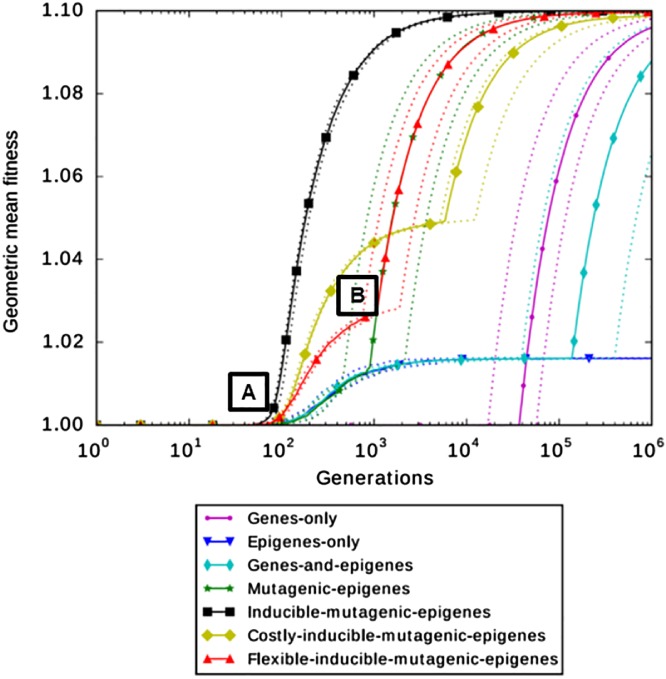
Timescales of adaptation in a model of genetic assimilation. Solid lines: median trajectory (in terms of evolutionary speed) of 10 independent simulations. Dashed lines: fastest and slowest trajectories, respectively. When a given curve lies above another one at a given timescale, the corresponding strategy wins over the other at that timescale (see text for details on strategies).

As qualitative results depend only upon the relative orders of magnitude of the parameters, results are illustrated with a single set of parameters (Fig. 4). However, the actual timescales of adaptation will depend on the relative biological parameters, that is the mutation rate (with a negative relationship), population size, the complexity of the selective force (with a negative relationship), and the steepness of the fitness landscape.

**Figure 5:**
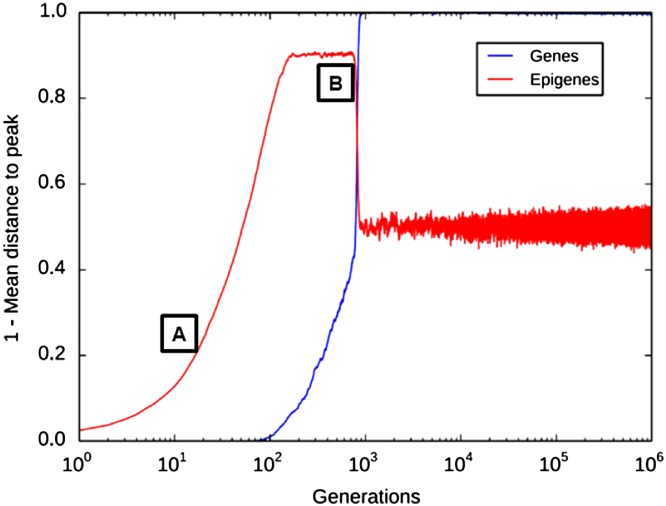
An example of genetic (blue) and epigenetic (red) dynamics from a single simulation with flexible‐inducible‐mutagenic‐epigenes (see text for details). This situation corresponds to the curve plotted with red triangles in Fig. 4.

We found that the relative timescale of adaptation depends strongly on the mechanistic links between genetic and epigenetic mechanisms (Fig. 4). In all strategies with both genes and epigenes, the initial increase in geometric mean fitness results from epigenes finding the peak and fit epigenes invading the population (point A in Figs 4 and 5). The second increase, if present, results from genes finding the peak and fit genes invading the population, at which point the selective pressure on epigenes is released, leading them to drift (point B in Figs 4 and 5).

We found that the *genes‐only* strategy (pink small circles, Fig. 4) usually wins over the *epigenes‐only* strategy (dark‐blue triangles, Fig. 4) over a timescale of 10^4^–10^5^ generations. This is because epigenes evolve faster, but have a higher mutation load.

The *genes‐and‐epigenes* strategy (light‐blue diamonds, Fig. 4) does as well as the *epigenes‐only* strategy in the short term (<10^5^ generations), but genes then take longer to find their peak. This is because the presence of fit epigenes slightly decreases the relative fitness of fit genes, increasing the probability that fit genes are lost by drift. Thus, having both genes and epigenes accelerates phenotypic adaptation (point A on Figs 4 and 5) but slows down genetic adaptation (point B on Figs 4 and 5). These results essentially replicate the simulations of Klironomos *et al*. (2013).

The *mutagenic‐epigenes strategy* (dark‐green stars, Fig. 4) outperforms the previous ones, with epigenes being mutagenic up to the point where epigenetically mutated fit genes invade the population (*t* ∼ 10^3^, i.e. two orders of magnitude earlier than with genes alone) at which point epigenes drift, leading to a higher proportion being in the non‐mutagenic stage, thus removing the mutation load on genes. This strategy represents our primary model of mutational assimilation, and replicates that of Jablonka & Lamb (1995). Note that simply increasing the baseline genetic mutation rate (possibly up to the epigenetic mutation rate) would not yield the same result, as this would not only increase the genes' ability to reach the peak, but also their mutation load (Pocheville & Danchin, 2017). Genetic adaptation by mutational adaptation thus depends on the articulation between epigenes and genes, i.e. on the fact that mutagenicity is induced by (here, fit) epigenes, meaning that once genes are fit, epigenes drift and lose their mutagenicity, decreasing the genetic mutation load.

With the *inducible‐mutagenic‐epigenes* strategy (black squares, Fig. 4) fit epigenes quickly reach the peak and fit genes, if any, never invade the population (except possibly by drift with an infinitesimal probability). Thus, adaptive induction of epigenes by the environment hampers genetic adaptation.

Genetic adaptation can be restored by the two last kinds of mechanisms explored here: a cost to the epigenetic mechanism (*costly‐inducible‐mutagenic‐epigenes*; green diamonds, Fig. 4), or a context dependence of their induction by the environment (*flexible‐inducible‐mutagenic‐epigenes*; red triangles, Fig. 4).

The *flexible‐inducible‐mutagenic‐epigenes* and *mutagenic‐epigenes* strategies lead to similar rates of genetic adaptation, where genetic adaptation is accelerated by a factor commensurate with the mutagenicity of epigenetic marks.

## IV. WHERE TO NEXT? EVOLUTIONARY IMPLICATIONS AND APPLICATIONS OF GENETIC ASSIMILATION

### (1) To what extent are mutations random?

**Figure 6:**
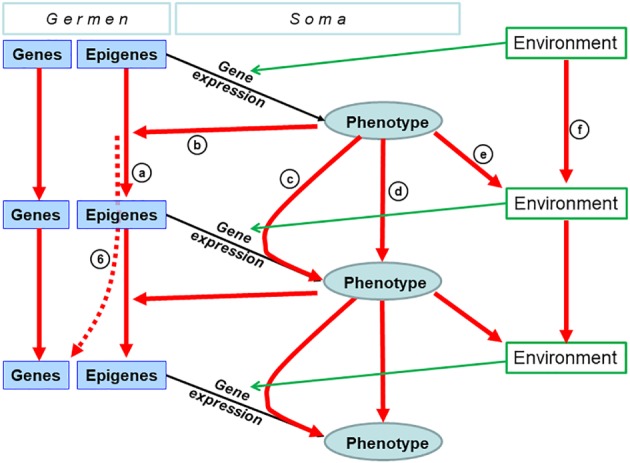
Inheritance according to the emerging inclusive vision of inheritance. According to this view, accumulation of inherited information can occur through a variety of pathways. Black arrows, development; green arrows, environmental effects; plain red arrows, pathways of intergeneration information inheritance; dotted red arrows, genetic assimilation that emerges over many generations as a consequence of the mutagenicity of heritable epigenetic marks. Arrow labels: (6) epigenetically facilitated mutational assimilation; (a) germline epigenetic inheritance; (b) soma to germline communication: (c) parental effect; (d) cultural inheritance; (e) niche construction; (f) ecological inheritance. Arrow 6 is strictly equivalent to arrow 6 in Fig. 2.

**Figure 7:**
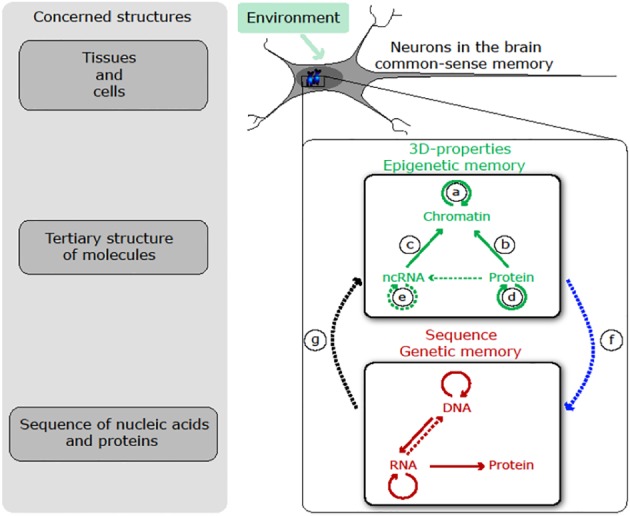
The various forms of biological memory. The red box is slightly modified from the central dogma of Crick (1970). As underlined by Crick, in this red box, molecular memory is engraved in the molecular sequences. Solid and dotted red arrows represent general and special information transfers respectively. The green box adopts the same formalism to depict epigenetic memory, which is contained in the 3D structure of macromolecules (Babbitt *et al*., 2016). Thus, chromatin can be seen as a gigantic prion. Here, we focus on a neuron, but this logic applies to any cell, including gametes. Arrow (a) illustrates that epigenetic marks are duplicated during DNA duplication, making them heritable within cell lineages. Arrow (b) represents the major role of proteins in chromatin structure. Arrow (c) represents the now well‐documented capacity of non‐coding RNAs (ncRNAs) to deeply affect chromatin structure. Arrow (d) represents the capacity of some proteins to transmit their configuration to other similar molecules as in prions. Arrow (e) represents that some ncRNAs are now suspected to self duplicate (Wang *et al*., 2017). The various potential pathways of genetic assimilation, here represented by arrow (f), are detailed in the central panel of Fig. 2, and the underlying evidence is reviewed in the text. Arrow (g) represents that the molecular sequence affects their 3D structure. Cell, epigenetic and genetic memories are the main processes of memory in unicellular organism. In multicellular organisms, cell and tissue memories can exist, and in organisms with brains common‐sense memory emerges from the structure and functioning of the central nervous system. According to the Inclusive Evolutionary Synthesis, all these forms of memory may participate in parent–offspring resemblance and hence in inheritance, and are thus open to natural selection.

The three elements reviewed above, (*i*) environmental change induces epigenetic change that interacts with TEs to produce germline genetic variation in specific loci, (*ii*) germline altered epigenetic patterns can be transmitted for many generations, and (*iii*) epigenetic marks are mutagenic, imply that the localization of genetic change is partly environmentally driven (Jablonka & Lamb, 1995, 2010; Noble, 2013). As illustrated by arrow 6 in Figs 2 and 6 and arrow (f) in Fig. 7, the environment somehow affects mutation rates in the very sections of DNA that were epigenetically affected by the environmental stressor. Which specific mutations occur is not determined by the environment itself, but the functional portions of the DNA molecule where the mutation rate changes have the potential to be guided by the environment *via* epigenetic marks, promoting genetic variation in these loci upon which selection can act, thus leading to epigenetically facilitated mutational assimilation. Thus, the evolutionary outcome is similar to that of directed mutations, although each mutation is still non‐directed [for a more complete discussion of this topic, see Pocheville & Danchin, 2017; for a discussion about randomness in biology see Merlin, 2010 and Razeto‐Barry & Vecchi, 2017]. Nonetheless, there are important differences. First, the model in Section III shows that such epigenetically facilitated mutational assimilation has the potential to accelerate the rate of the genetic engraving of recently acquired adaptations. Second, the proposed mechanism has the potential to considerably diminish the impact of deleterious effects linked to mutation load.

### (2) The two timescales of epigenetics: from development to a mutational engine of genetic assimilation

Epigenetic marks can thus act at two contrasting, yet complementary, timescales. In the short term, they adaptively fine‐tune the expression of genes potentially modulating phenotypes to local conditions in the exposed generation (plasticity). At the across‐generations timescale, they affect the mutability of the very same DNA sequences, hence affecting evolutionary changes in the relevant functional DNA sequence over generations (Rey *et al*., 2016). These two timescales are illustrated in *C. elegans* where neuron‐produced double‐stranded RNAs generate both intragenerational epigenetic silencing in somatic cells, and intergenerational epigenetic silencing in germ cells (Ashe *et al*., 2012; Devanapally *et al*., 2015; Klosin *et al*., 2017; review in Vastenhouw *et al*., 2006; Remy, 2010; Minkina & Hunter, 2018). Such unsuspected soma‐to‐germen communication (Sharma, 2015) in animals provides a potential mechanistic basis for the arrows converging towards epigenetics in Fig. 2. In plants, as there is no soma–germen separation because reproductive cells differentiate from somatic cells, gametes derive from cells that were exposed to environmental stressors so that soma to germen communication might be much more common. Figure 2 thus describes a ‘mutational engine’ targeting the genes that participate in the accommodation to specific environmental changes, eventually fine tuning ‘the timescale of their own heritable variation to match the timescale of the acting selective pressure’ (Rando & Verstrepen, 2007, p. 656). The relay among inheritance systems that we propose here for shifts among inheritance systems and for which we provide a simple model suggests that epigenetically mediated environmentally driven mutations can directly accelerate the engraving of the variability of the functional traits into the DNA sequence.

### (3) Inheritance according to the emerging inclusive evolutionary synthesis

Weismann's view was that acquired characters cannot be inherited (Fig. 1; Griesemer & Wimsatt, 1989). After the Modern Synthesis, the dominant view was that heredity requires only the transmission of the gene (which is now most often equated to the DNA sequence), implicitly neglecting the evolutionary potential of non‐genetically inherited variation. Obviously, Darwin's view of natural selection did not require inheritance to be limited to DNA sequence transmission. Similarly, classical (first half of the 20th century) and modern population genetics do not assume inheritance to be limited to DNA sequence transmission.

On the contrary, within the inclusive evolutionary synthesis, heredity also involves several other interconnected information pathways (labelled arrows in Fig. 6). Several theoretical studies have revealed the potential impact of non‐genetic inheritance on phenotypic and genetic adaptation leading populations to equilibria that would be unreachable with gene‐only inheritance (Jablonka & Lamb, 1995; Lachmann & Jablonka, 1996; Heyer *et al*., 2005; Klironomos *et al*., 2013; Townley & Ezard, 2013). Similarly, our model suggests that introducing mutagenic epigenetic marks may accelerate genetic adaptation by a factor similar to the rate of mutagenicity [around 2 × 10^4^ for methylcytosine (Jones *et al*., 1992; Schmutte *et al*., 1995; Gorelick, 2003)], provided that the concerned environmental change persists over a timescale at least equivalent to that of the multigenerational scale of the epigenetically facilitated mutational assimilation. At a macro‐evolutionary scale, several studies have suggested that the epigenetic differentiation of populations may affect speciation, as for instance in hominids (Gokhman *et al*., 2014), fish (Smith *et al*., 2016) and Darwin's finches (Skinner *et al*., 2014). With this perspective, it appears that Darwin's view of inheritance was closer than the Neo‐Darwinism vision of inheritance to the inclusive view of inheritance that we discuss here, whereby inheritance mechanisms of very different natures interact to produce a single mechanism that generates the potential for populations to evolve under natural selection.

### (4) Non‐genetic inheritance and the central dogma of molecular biology

Another interesting twist in this vision concerns the central dogma of molecular biology, which was formulated in terms of sequence (i.e. primary structure) of macromolecules (red box in Fig. 7; Crick, 1970). Some non‐genetic inheritance systems are encoded into the tertiary (three‐dimensional) structure of molecules (green box of Fig. 7) or at even higher levels of organisation (cell and tissues). In epigenetics, for instance, memory is encoded into the chromatin structure, which results from a variety of molecular memory systems involved in the tertiary structure of molecules (Fig. 7) that determine their accessibility for transcription. The causal links between the 3D structures of DNA, RNA and protein differ sharply from those known at the sequence level that are at the heart of the central dogma (compare the red and green boxes in Fig. 7). In cultural inheritance, this memory is carried out at an even higher level of organization, reaching that of the neurons within the brain tissues (Fig. 7).

Interestingly, by affecting germline epigenetic marks, the environment may eventually modify the DNA sequence over the course of generations [arrow (f) of Fig. 7, and arrow 6 in Figs 2 and 6]. However, by unfolding at a multigenerational timescale, mutational assimilation, and more generally inclusive inheritance, does not violate the central dogma at the developmental timescale as formulated by Crick (1970): to our knowledge, the information engraved in the protein sequence has never been shown to be able to affect the corresponding DNA sequence.

### (5) Validating this hypothesis: a call for empirical data

Our review also underlines the lack of data causally linking epigenetic marks to mutation rates. In particular, such links may vary according to mutation type. In both germline and somatic cells, regions of closed chromatin show higher levels of base substitutions (Schuster‐Boeckler & Lehner, 2012), while higher levels of insertions, deletions and sequence substitutions occur in regions of open chromatin (Makova & Hardison, 2015). This clearly indicates that links between epigenetic marks (a major determinant of chromatin accessibility) and mutation rates should distinguish among these various types of mutations. We also need to understand chromosome organisation in the germline better as this may strongly affect mutation rates (Schuster‐Boeckler & Lehner, 2012). In particular, it will be necessary to design experiments able to capture variation in epimutation and mutation rates according to the epigenomic context of the genes or group of genes.

Documenting these links constitutes a major challenge, but is central to validate empirically the existence of the relay among inheritance systems that we propose here. For that goal, experimental evolution, or selection experiments coupled with high‐throughput sequencing and epi‐sequencing (bisulfite for instance) as in Kronholm *et al*. (2017) represent particularly promising approaches. However, in contrast with the usual mechanistic approaches at the scale of one or a few generations, epigenetically facilitated mutational assimilation may only be detected at the larger scale of many generations. Consequently, although the proposed epigenetically facilitated mutational assimilation may greatly accelerate genetic evolution (see Section III), its detection may require experiments over unusually long timescales of many generations. Biology will thus need to adapt to such timescales, maybe finding inspiration from disciplines such as astrophysics. Overcoming such challenges will be necessary to unravel these suspected inheritance mechanisms with potentially momentous implications in evolution, medicine and conservation in general (see below).

### (6) Conceptual implications: the need for more theoretical approaches

Our goal is to help to integrate scientific approaches at the infra‐ and supra‐individual levels into a unified view accounting for the fact that the various inheritance systems interact as runners in a relay race, handing over heritable information between stages, hence potentially matching the timescales of inheritance with those of environmental variation. What we need now are models of informational dynamics integrating such a relay at the ecological and evolutionary timescales. Comparing Figs 1B and 6 suggests that this might substantially change the properties of the equations formalizing informational dynamics across generations, although this remains to be explored experimentally and theoretically.

Although several theoretical studies have tackled questions such as the interaction between epigenetic and genetic evolution (Hinton & Nowlan, 1987; Jablonka & Lamb, 1995; Lachmann & Jablonka, 1996; Pal, 1998; Pal & Miklos, 1999; Klironomos *et al*., 2013) we are still far from possessing an integrative theoretical framework of the interactions among plasticity, inheritance and evolution. In our simple model, we revisit some of these models to analyse the potential impact of mutational assimilation at the evolutionary timescale. This shows that the relay among inheritance systems may considerably accelerate (but also, depending on biological circumstances, slow down) the genetic encoding of adaptation. One outcome of the proposed mechanism of epigenetically facilitated mutational assimilation is that the mutation rate should vary among genes according to whether they are involved in the adaptation to the specific environmental change. Future models will thus need to incorporate some variation in local mutation rates to explore the impact of the proposed mechanism of assimilation. Furthermore, our and previous models suggest that incorporating non‐genetic inheritance should provide us with models where not only non‐genetic and genetic inheritance systems are unified, but also developmental processes (Danchin & Pocheville, 2014; Pocheville & Danchin, 2015). This implies that events at the timescale of a lifetime can still be drivers of evolution. This line of thought, if validated, would go against the usual (and practical) timescale separation between proximate and ultimate processes (Mayr, 1961; Laland *et al*., 2011) as assumed in models since the Modern Synthesis (Pocheville, 2010; Braun, 2015).

### (7) Medical implications

Inclusive inheritance has paramount medical implications by allowing the study of the various components of the inheritance of so‐called ‘genetic disorders’, which in fact may be substantially inherited non‐genetically (Holliday, 1987; Hales & Barker, 2001; Gluckman *et al*., 2009; Brookfield, 2013; Danchin, 2013; Trerotola *et al*., 2015). Concerning the hypothesis of epigenetically facilitated mutational assimilation, it should be noted that epigenetic change has long been suspected to cause the early stages of tumour genesis, as regional changes in epigenetic marks, and particularly DNA methylation, often precede cancers (reviews in Gonzalgo & Jones, 1997; Plass & Soloway, 2002; Sawan *et al*., 2008). As stated above, genomic landscape features are suspected to act in synergy to explain variation in mutation rates (Makova & Hardison, 2015), and the mutations that underlie tumour genesis may actually represent a consequence of disrupted epigenetic states rather than the initial cause of cancer (Sawan *et al*., 2008). If the claim that it is the mutagenicity of epigenetic marks that generates mutations causing cancers (Gonzalgo & Jones, 1997; Plass & Soloway, 2002; Sawan *et al*., 2008; Makova & Hardison, 2015; Polak *et al*., 2015) proves to be true, this would constitute an example of epigenetically facilitated mutational assimilation unfolding during an individual organism's lifetime. This would imply that applying our model and Fig. 2 to generations of cells within an organism could be relevant to the study of the initial stages of cancer.

It is beyond the scope of this paper to review all the medical implications of inclusive inheritance, but if our proposed mechanism is true, it may well be that medical research should move away from a purely genocentric vision of inheritance. Adopting a more open‐minded vision of inheritance may allow the discovery of new therapies, for instance, for cancer (Plass & Soloway, 2002; Sawan *et al*., 2008; Mack *et al*., 2014; Versteeg, 2014) and open new avenues to study epigenetically facilitated mutations.

### (8) Towards an inclusive evolutionary synthesis

Although the discovery of genetics dramatically improved our understanding of inheritance and evolutionary biology, it had the downside of considerably narrowing our vision of inheritance and evolutionary biology, hence pushing the role of other inheritance mechanisms out of view. One consequence of this is that established textbook examples of genetic determinism have later been revealed to be caused by epigenetic variation (Cubas *et al*., 1999; Wang *et al*., 2017). Many examples are presented in Table 1. This review attempts to help to reopen the vision of inheritance to develop an integrative theory accounting for recent empirical discoveries and to include how the various mechanisms of inheritance complement each other. We thus document potential mechanisms of mutational assimilation imposed by the pace of environmental change and for which epigenetic inheritance plays the role of a hub. Our ambition is to extend our vision of inheritance in order to make it more inclusive than the mainstream genocentric vision that is most often taught to students. The possibility of a relay among inheritance systems may change our perspective on the causes of evolution, because diversity does not result solely from random mutations, but also partly from environmentally driven variation (Lindquist, 2011). In this framework, the environment emerges as a generator of diversity upon which evolution can act, a process in which epigenetics could play a major evolutionary role. Although first viewed as a mechanism of development and plasticity, epigenetics now emerges as a hub between development, ecological change and evolution. Epigenetics has the potential to kick‐start or stabilize adaptive evolution in the long term. These considerations bring important arguments for adopting a more‐inclusive perspective of heredity within the evolutionary synthesis by adding these emerging and interacting processes of non‐genetic inheritance. Such a more‐inclusive vision of evolution has the potential to provide more‐complete explanations of the biodiversity that we observe in nature.

## V. CONCLUSIONS

(1) We revisited the diverse mechanisms of inheritance according to relevant timescales. We suggest the existence of shifts between inheritance mechanisms that produce a relay in information encoding that enables a lineage to match the pace of environmental change.

(2) We reviewed the evidence for such potential shifts of information. Epigenetics appears as a major hub in this relay towards genetics, linking non‐genetic germline inheritance with genetic inheritance in a form of epigenetically facilitated mutational assimilation.

(3) A theoretical model suggests that such mutational assimilation may considerably accelerate (or slow down) the genetic encoding of initially non‐genetically inherited adaptations, by a factor commensurate with that of the mutagenicity of epigenetic marks.

(4) This view of inheritance has major practical implications, and opens the way for new studies at scales ranging from the molecular to the population levels.

## VI. ACKNOWLEDGEMENTS

Aurélien Pocheville greatly helped in programming the model. At the time of writing, A.P. had a postdoctoral fellowship at the Centre for Philosophy of Science, University of Pittsburgh. Philippe Huneman helped in discussing several points in this review. We are thankful to the CNRS InEE pluridisciplinary research network (RTP) in Epigenetics in Ecology and Evolution (3E) for insightful discussions. This work was supported by the ‘Laboratoires d'Excellences (LABEX)' TULIP (ANR‐10‐LABX‐41), as well as ANR funded Toulouse Initiative of Excellence ‘IDEX UNITI’ (ANR11‐IDEX‐0002‐02). E.D., S.B. and B.P. were supported by the Soc‐H^2^ ANR project (ANR‐13‐BSV7‐0007‐01) to E.D. B.P. was also supported by the CAPA ANR project (ANR‐13‐JSV7‐0002), and is part of a project that received funding from the European Research Council (ERC) under the European Union's Horizon 2020 research and innovation program (ERC consolidator grant agreement No ERC‐CoG681484‐ANGI). A.P. was supported by the Templeton World Charity Foundation, Inc. (grant TWCF0242). The opinions in this publication are those of the authors and do not necessarily reflect the views of the Templeton World Charity Foundation, Inc.

## Supporting information


**Table S1.** A description of published models of non‐genetic inheritance and genetic assimilation.
**Table S2.** Description of the model parameters.
