# Supplementary material for: Epigenetically facilitated mutational assimilation: epigenetics as a hub within the inclusive evolutionary synthesis
Source: Biol Rev Camb Philos Soc. 2018 Sep 24;94(1):259–82. doi: 10.1111/brv.12453 (PMC6378602; doi:10.1111/brv.12453)
Supplement: Supplementary file 1 — Table S1. A description of published models of non‐genetic inheritance and genetic assimilation. Table S2. Description of the model parameters. [file BRV-94-259-s001.docx]

# Supporting information

**Table S1.** A description of published models of non-genetic inheritance and genetic assimilation. Unless otherwise specified, models are generally non-sexual and generations non-overlapping (although survival and reproduction are generally not explicitly distinguished), rates of forward or backward epigenetic mutations are identical, and variation is blind (i.e. does not increase fitness) even when induced by the environment. Several other notable dual-inheritance models are not included (e.g. Boyd & Richerson, 1985; Laland *et al*., 1999; Lehmann, 2008).

|  | **Brief description** | **Selection of results** | |
| --- | --- | --- | --- |
| **Hinton & Nowland (1987)** | An organism is represented by a neural net, the connections of which can be determined by learning trials and/or genes. ■ There are 20 genes, each with three alleles: ‘1’ (connection present), ‘0’ (connection absent) and ‘?’ (can be switched by learning). Each allele specifies 20 potential connections. Initial probabilities are *p*(?) = 0.5 and *p*(1) = *p*(0) = 0.25. ■ The phenotype is the set of connections in a neural net. Correct sets of connections are frozen. ■ Fitness is added only if the net is in the right configuration. The probability of being a parent is proportional to 1+19*n*/1000, where *n* is the number of learning trials that remain after the organism has learned the correct set of connections. ■ There are 1K organisms, and 1K learning trials per organism per generation, 50 generations per simulation. ■ Sex is represented (diploid, mating at random). ■ Variation is blind, not induced by the environment (learning is random, but the configuration is frozen if correct). ■ The model is a simulation (genetic algorithm). | Learning alters the search space. Evolution is much faster even if no information flows from acquired characteristics to inherited characteristics. ■ There is very little selective pressure to genetically specify the last correct connections (a few learning trials suffice to find them). ■ Far less than the 2^20^ organisms needed for a pure genetic search are produced. ■ The peak is never reached by a pure genetic search; even if the right genotype is discovered, it would not be easily transmitted. | |
| **Kirkpatrick & Lande (1989)** | Individuals are characterized by Mendelian and non-Mendelian heritable characters (the linear combination of which adds to fitness), plus a direct effect of parents on fitness. The model considers only vertical inheritance. Two versions: one-locus and multi-locus. ■ Variation is blind. ■ Population size is indefinite. ■ Results are mostly analytical. | Time lags occur in the dynamics. ■ Maternally inherited traits respond to current and previous selection at the same time. ■ Time lags are temporary, oscillations dampen. ■ Ultimate evolutionary rate is reached only asymptotically (but immediately with only Mendelian inheritance). ■ Time lags possibly occur for as many generations as the number of characters in the multi-locus version. | |
| **Jablonka & Lamb (1995)** | A gene can alternate between two states (G1, G2), with a mutation rate (as well as its activity) determined by the state of an epigenetic mark. There are two states of epigenetic marks, m1 and m2; such that: m1 silences the gene and is non-mutagenic, and m2 makes the gene active and is mutagenic. ■ Basal µ_g_ = 10^–6^, and when the mark is mutagenic µ_g_ = 10^–4^. ■ Transitions between epigenetic states can be spontaneous  (10^–2^) or induced by the environment towards the good state (100%). ■ Selection can be neutral, or small (1% for G2). ■ Results are obtained by simulation. | The frequency of the initially rare allele increases as a result of mutational supply; even when there is no selection (as a result of different mutational equilibrium). ■ This model is similar to our cases of ‘mutagenic epigenes’ but is analysed rather in terms of dynamics of frequencies rather than explicit timescales of adaptation. | |
| **Jablonka *et al*. (1995)** | Individuals are described by a single variable P with values P1 and P2. Environment oscillates between states E1 and E2. Both can induce and positively select phenotypes P1 and P2, respectively. Oscillations can be: (1) random with *p*(transition) = 0.5; (2) ‘temporally patchy’ (*p* > or < 0.5); (3) strictly periodic, with *T* = 2*n*. ■ Three strategies are considered: (1) ‘pure genetic’ (changes from and to P1and P2 occur at rate µ < 10^–5^, induction rate is *v* = 0); (2) ‘plastic’ (the phenotype is induced towards the good phenotype at rate *v* = 1.0, i.e. always – note there is a time lag between induction and selection so that the environment can change before selection); (3) ‘carry-over’ (phenotypes are transmitted to the progeny at rate 1–*v*, with induction rate 0 < *v* < 1). ■ Results are obtained by analysis and simulations (1K generations). | In the random environment, a bet-hedging strategy wins; by construction it can be achieved only with strategy (3) (it is thus not so much the carry-over effects which are selected in this environment, but rather the ability to perform bet-hedging). ■ In the patchy environment, if the environmental transition rate is close to random, the carry-over strategy wins; if it is under or above a certain threshold, the plastic or pure-genetic strategies win. ■ In the periodic environment, the plastic strategy wins (except if *n* = 1, where it is out-of-phase, or *n* = 2, where it is on a par with the pure genetic strategy). | |
| **Lachmann & Jablonka (1996)** | The model is identical to the ‘strictly periodic’ model above. ■ Individuals are described by a single variable P with values P1 and P2. Environment oscillates between states E1 and E2. Both positively select phenotypes P1 and P2, respectively. Oscillations are strictly periodic, with cycle length *n* = 2–100. The case of phenotypic induction by the environment is also considered. ■ Population size is indefinite. ■ Results are analytical. | When transition is random (non-induced), the optimal transition rate is approximately 1/*n*. Selection for optimal transition is strong (order of √(*s*)). ■ When phenotypic transition is induced towards fitting, without a lag in phenotypic response, the optimal transition rate is *v* = 1. ■ See also Jablonka *et al*. (1995). | |
| **Pal (1998)** | Individuals are characterized by their genotype, with two independent loci determining the mean of a trait and its variance, respectively. ■ The environment is assumed constant. Centred environmental micro-fluctuations induce centred (blind) phenotypic variations, the variance of which is genetically determined (by a plasticity locus). ■ Phenotype is Gaussian around a genetically determined mean. ■ The fitness landscape is single-peak (Gaussian). ■ Three strategies are examined: (1) gene only (2) plastic with the frequency of phenotypes being genetically determined; (3) with epigenetic memory (range: one to infinity). ■ Population size is indefinite. ■ Results are analytical. | The dual inheritance of the character can be advantageous for the genotype when the character is far from the peak. Close to the peak, suppressing phenotypic variation is more advantageous to the genotype. This leads to genetic assimilation. [This analytical model encompasses the later simulation models by Klironomos *et al*. (2013) and ours except for cases of mutational assimilation.] | |
| **Pal & Miklos (1999)** | This model reiterates that of Pal (1998), but adds the case of a bimodal Gaussian fitness peak, treated by simulations (population size 1K–4K, time 10^2^–10^9^). | The variation in (heritable) epigenetic marks reduces the valleys between two adaptive peaks for genes (phenotypes might be on peaks even if the gene is not). Peak shift might be induced by environmental change or genetic drift. ■ Drift-induced shift is facilitated even if epigenetic marks are not heritable. ■ The expected time of peak shift for the genes is lower when epigenetic variability can evolve. | |
| **Lande (2009)** | Individual phenotype is a linear combination of a fixed genetic component, and a plastic component determined by (i.e. proportional to) the value of the developmental environment (i.e. there is a time lag before selection). ■ The fitness landscape is single-peak (Gaussian). ■ The population is at first adapted and the phenotype canalized, then the environment suddenly changes with a magnitude greater than normal fluctuations. ■ Population size is indefinite. ■ Results are analytical. | The initial optimal plasticity is proportional to the time lag between development and selection. ■ The sudden environmental change causes a fitness drop, with rapid phenotypic accommodation due to plasticity. ■ Adaptation then occurs in two phases: (1) evolution of plasticity; (2) canalization by slow genetic assimilation. | |
| **Helanterä & Uller (2010)** | Classify effects of inheritance mechanisms in terms of the Price equation. | The Price equation framework suggests that the previous classification of inheritance systems into genetic, epigenetic, behavioural and symbolic (Jablonka & Lamb, 2005) does not fully capture evolutionary differences and similarities between inheritance systems. | |
| **Day & Bonduriansky (2011)** | Analyse and simulate various models of inheritance (genetic, epigenetic) in terms of the Price equation. ■ Individuals are characterized by one genetic locus (two discrete values or a quantitative character, e.g. breeding value) and one epigenetic (or cultural) locus (two epigenetic values or a quantitative character, e.g. state of cellular machinery). ■ Phenotype is determined according to various models (in the form of sums of genetic and epigenetic effects, from current or previous generations). Fitness is determined by the phenotype. ■ Diverse scenarios are considered, including non-transmissible environmental noise, maternal effects, indirect genetic effects, transgenerational epigenetic inheritance, RNA-mediated inheritance, and cultural inheritance. ■ Overlapping generations are explicitly considered. ■ Results are mostly analytical, simulations are also provided. | Similar to Kirkpatrick & Lande (1989) for maternal effects and indirect genetic effects. ■ As expected, gene-silencing silences selection. RNA inheritance can modify the strength (but not the sign) of selection (RNA behaves as a phenotype). ■ As expected, there is a partial, transient decoupling of the dynamics of the phenotype and the genotype (i.e. time lag). | |
| **Klironomos *et al*. (2013)** | Individuals are characterized by one DNA sequence (characterized by a set of variables *g*_1_, *g*_2_, … *g*_k_) and one epigenetic system (described by set of variables *e*_1_, *e*_2_, , … *e*_l_) (with *k* = l = 10). ■ Mutations are blind and rare (µ_g_ = 10^–6^, µ_e_ = 10^–4^). ■ Fitness is a function of genes and epigenes: *w*_genetic_(*g*_1_, *g*_2_, …) and *w*_epigenetic_(*e*_1_, *e*_2,_ …), *W* = max(*w*_genetic_, *w*_epigenetic_). ■ Two types of fitness landscape: (1) single-peak, *w*_genetic_ = 1.5 if *g*_1_ = *g*_2_ = ... = 1 (on peak) and 0.1 otherwise; idem for *w*_epigenetic_; (2) multi-peak, each fitness is drawn independently from a Gaussian distribution of mean 1.1 and std dev 0.25 (rugged landscape with many peaks and valleys). ■ Simulations are run using a standard Wright–Fischer dynamics (number of individuals = 1000, number of generations ~ 10^6^). | Single peak: epigenes adapt quickly (genetic polymorphism: genes evolve neutrally), and are eventually replaced by on-peak genes with a lower mutation load (epigenes evolve neutrally). ■ Multi-peak: similar, with a series of adaptive steps. ■ The gene-only strategy gets stuck at a local maximum. ■ The simulation results closely replicate Pal’s (1998) analysis. | |
| **Nishikawa & Kinjo (2018)** | The population consists of *N* individuals, each having *L* = 20 genes. Mutation takes value 0 (wild) or 1 (mutant). At one locus genetic contribution can be 1 (advantageous) or –1 (disadvantageous). ■ Two models of phenotypic expression are considered: (1) conventional model, the phenotype is a linear combination of the genetic contributions (initial standard deviation < 0.45) and an environmental effect, which is a random, non-inherited variable (std dev = 0.5); (2) cooperative model, same model, but the standard deviation of the environmental effect (now named ‘epigenetic effect’, still non-inherited) is larger (3 instead of 0.5). ■ The model is implemented in a genetic algorithm. Initially, the fitness effect is 1 for the first 10 loci and –1 for the next 10. ■ Individuals mutate (once and for all) with a rate per gene = 10^–2^. No mutations are generated subsequently. ■ At each generation 2*N* pairs of individuals mate at random with sexual reproduction (crossover at a random site). 4*N* offspring are produced; parent individuals are discarded. ■ For each new individual, an environmental (model 1) or epigenetic (model 2) value is randomly generated. Individuals whose phenotype is above a threshold (here set to 5) are selected with probability 1, those with a negative phenotype are discarded with probability 1, those in between are selected with probability *q* = 0.15. ■ The threshold mimics a qualitative change. ■ Population size (initial and maximum) is set to 10^5^ (excess individuals are removed at random). | | In the conventional model, populations disappear rapidly (15 generations). ■ In the cooperative model, the population adapts on a commensurate timescale. The epigenetic contribution (i.e. random plasticity, in this model) increases rapidly, before gradually decreasing, while the genetic contribution steadily increases, reflecting genetic assimilation. ■ In both models genetic variation pre-exists the selective event and is revealed by crossovers at each generation. |
| **Kronholm & Collins (2016)** | Adaptive walk, simulated with an agent-based model. Three steps: (1) individuals mutate, with epigenetic mutations (frequent and small) or genetic mutations (rarer and bigger); (2) individuals reproduce according to their fitness, modelled as a negative exponential of the distance to optimum; (3) reverse epigenetic mutations can occur. ■ Individuals are characterized by one genetic state and one epigenetic state, each taking values in an hypersphere of dimensionality 25 (phenotype = distance to centre of the hypersphere). Epigenetic and genetic mutational effects are modelled as vectors random in angle and magnitude. Epigenetic effects are a subset of genetic effects. ■ Fitness effects are notably arbitrary (fitness decreases from optimum as a Gaussian function, i.e. there is stabilizing selection when at optimum). ■ Simulations are run for 20K generations, with initial population size = 1K. Reverse epigenetic mutation is explicitly distinguished from forward mutation. ■ Results are obtained by simulations. | | Epigenetic mutations can have various effects on adaptation. They can speed up the initial stages of adaptation but also reduce final population fitness (epigenetic fitness effect slightly smaller than those of genetic mutations), or slow early adaptation while allowing fine-tuning (higher fitness) in the late stage (small epigenetic fitness effects), or slow adaptation and result in lower fitness (when they have the same distribution of fitness as genetic mutations, because they have a higher mutation load). |
| **Present model** | Individuals are characterized by one genetic variable (7 bits) and one epigenetic variable (7 bits). ■ Adaptive landscape is single-peak (*g*_1_ = *g*_2_ = … = *g*_7_ = 1 AND/OR *e*_1_ = *e*_2_ = … = *e*_7_ = 1); with *w*_peak_ = 1.1 and *w*_off-peak_ = 1. *W*_individual_ = max(*w*_genes_, *w*_epigene_), i.e. there is full redundancy between genetic and epigenetic factors. ■ Genetic variation is always blind (µ_g_ = 10^–4^ per sequence), epigenetic variation is generally blind (µ_e_ = 10^–1^ per sequence) except when epigenes are induced by the environment (see below). ■ A set of strategies are explored: (1) genes only (no epigenetic variable); (2) epigenes only (no genetic variable); (3) genes and epigenes (each variable mutates independently); (4) mutagenic epigenes: mutation of genes is increased by a factor *n**10^2^, where *n* is the number of epigenetic bits = 1; (5) inducible (mutagenic) epigenes: when epigenes are mutated, they are mutated towards fitness (0 → 1 and 1→ 1); (6) costly (inducible, mutagenic) epigenes: *w*_peak epigene_ = 1.05; (7) flexible (inducible, mutagenic) epigenes: inducing epigenes ceases when individual is at peak (either epigenetically or genetically). ■ Simulations are run for 10^6^ generations, with *N*_max_ = 1000 (random removal of surplus individuals if present). ■ Results are obtained by simulations. | | Randomly mutated epigenes and environmentally induced (towards fitness) epigenes accelerate phenotypic adaptation but generally slow down or prevent genetic adaptation [similar to Pal (1998), Klironomos *et al*. (2013), and Kronholm & Collins (2016) in their case of similar fitness effects]. ■ Genetic adaptation occurs and is accelerated if epigenes are mutagenic [mutational assimilation, similar to Jablonka & Lamb (1995)], except if they are induced towards fitness. This acceleration is restored if the induction is flexible (i.e. induction occurs only when individual is off-peak) or if the epigenes are costly [their maximum fitness is below the maximum fitness for genes, a situation comparable to Kronholm & Collins (2016) case of slightly lower fitness effects]. |

**Table S2.** Description of the model parameters.

| **Parameter** | **Value** |
| --- | --- |
| Population size | *N* < 1K individuals |
| Number of bits of the gene | *k* = 7 bits; each bit takes value 0 or 1 |
| Number of bits of the epigene | l = 7 bits; each bit takes value 0 or 1 |
| Fitness landscape for the gene | *w*gene peak = 1.1 (all genetic bits are 1s); *w*gene off-peak = 1 (otherwise) |
| Fitness landscape for the epigene (general case) | *w*epigene peak = 1.1 (all epigenetic bits are 1s); *w*epigene off-peak = 1 (otherwise) |
| Fitness landscape for costly epigenes (for individuals with costly epigenes) | *w*costly epigene peak = 1.05; *w*costly epigene off-peak = 1 |
| Fitness function for the individual | *w*individual = max[*w*gene, *w*epigene] |
| Mutation rate for the gene | μgene = 10^–4^ (per sequence) |
| Mutation rate for the epigene | μepigene = 10^–1^ (per sequence) |
| Induction rate by the environment (for individuals with inducible epigenes) | *r* = 1 (i.e. 100% of mutated epigenetic bits turn into 1s) |
| Mutagenicity of epigenes (for individuals with mutagenic epigenes) | μgene new = μgene*10^2^ **p* (where *p* is the proportion of epigenetic bits which are 1s) |
